# Comprehensive Evaluation of Quality and Differences in *Silene viscidula* Franch from Different Origins Based on UPLC-ZENO-Q-TOF-MS/MS Compounds Analysis and Antioxidant Capacity

**DOI:** 10.3390/molecules29204817

**Published:** 2024-10-11

**Authors:** Shaohui Zhong, Dezhi Shi, Yingxue Fei, Chengchao Wu, Jinyao Zha, Fangqi Lu, Yunyu Zhang, Jing Ji, Taoshi Liu, Jianming Cheng

**Affiliations:** 1 College of Pharmacy, Nanjing University of Chinese Medicine, Nanjing 210023, China; 20220799@njucm.edu.cn (S.Z.); 20210720@njucm.edu.cn (D.S.); 20230878@njucm.edu.cn (Y.F.); 20220949@njucm.edu.cn (C.W.); 20230874@njucm.edu.cn (J.Z.); 20220968@njucm.edu.cn (F.L.); 830113@njucm.edu.cn (Y.Z.); jier4522@163.com (J.J.); 2 Jiangsu Province Engineering Research Center of Classical Prescription, Nanjing 210023, China

**Keywords:** *Silene viscidula* Franch, difference in origin, UPLC-Q-ZENO-TOF-MS/MS, chemical compounds, antioxidant activity

## Abstract

*Silene viscidula* Franch is mainly produced in southwest China. The region has a vast area and rich climate, which has an impact on the quality of the plants due to the differences in distribution between the origins. There is a lack of systematic research on its chemical compounds in the existing literature, and fewer studies have been reported for the active compounds of this plant. Therefore, high-resolution liquid mass spectrometry was used in this study. Sixty batches of *Silene viscidula* Franch samples from twenty origins in three provinces were analyzed for compounds. A database of chemical compounds of *Silene viscidula* Franch was established through node-to-node information in the GNPS molecular network, as well as literature records. The ion fragmentation information obtained was compared with the literature data and analyzed and identified by importing the mass spectrometry software PeakView 1.2. Then, the MarkerView *t*-test was applied to analyze and identify the compounds of *Silene viscidula* Franch from different origins. Afterwards, the antioxidant activity of *Silene viscidula* Franch from different origins was preliminarily evaluated using DPPH and ABTS free radical scavenging assays. The results showed a total of 78 compounds, including 34 steroids, 14 triterpenoid saponins, 30 flavonoid glycosides, and other classes of compounds, such as alkaloids. The cleavage patterns of steroids, triterpenoid saponins, and flavonoids in positive-ion mode were also summarized. Based on the *p*-value of the *t*-test (*p* < 0.05), 29 differential compounds were screened out. The relative contents of saponins and steroidal compounds in these samples were found to be associated with antioxidant activity. This study provided a preliminary reference for the establishment of a comprehensive evaluation system for the quality of *Silene viscidula* Franch.

## 1. Introduction

*Silene viscidula* Franch (SF) was also known as WaCao in China. The plant was recorded for the first time in Yunnan Materia Medica [1], used as a medicine from the root part of the plant. It has pharmacological effects, such as analgesic, purging heat, resolving phlegm, diuretic, etc. [1]. It is widely used in the treatment of bruises, rheumatism pain, traumatic bleeding, sores, boils, poisons, etc. [1]. Currently, the research on SF mainly contains chemical compounds, such as saponins [2], steroids [2], flavonoids [2], and polypeptides [3].Its pharmacological effects are therapeutic for type 2 diabetes [4], cervical cancer [5], and other diseases, as it can significantly lower blood sugar and inhibit the growth of cancer cells. In terms of analgesia, it has a good inhibitory effect on inflammatory pain [6], and can significantly increase the pain threshold. SF is rich in chemical compounds and has a wide range of pharmacological activities, which has an important application prospect. However, it is still not included in the national standard, and the research on the compounds and quality standard of SF is not yet perfect, so the systematic research on its chemical compounds and quality analysis is of great significance.

China contains rich plant resources, among which SF comes from many provinces in the southern region of China (including Yunnan, Sichuan, Guangxi, and other provinces). These SF samples come from a number of regions where the climatic abundance varies considerably, so there will be some variation in the quality of SF from different origins. These SF plants have been used for a long time in the local populations with a wide range of application scenarios. Therefore, there is a great potential for the theoretical research and the development of the practical application of this drug in the clinic. In this study, the chemical compounds of SF from different origins were systematically analyzed and characterized. The quality differences in SF were evaluated from the differences in chemical compounds combined with the differences in antioxidant capacity.

The characteristic compounds of SF are currently saponins and steroids, which have been found to have significant pharmacological activity [2,7]. However, rapid and accurate identification is difficult due to the influence of control products and literature records of the compounds of SF. In this regard, in order to systematically establish and clarify the chemical compounds of SF, the UPLC-Q-ZENO-TOF-MS/MS technique was used in this study for rapid scanning and identification analysis. This method [8] has the advantages of short time and high sensitivity, which can be used for separation and analysis of complex samples and structure identification. The mass spectrometry data were imported into GNPS molecular network analysis [9,10]. This network can enrich similar compounds and form a network of node sets for rapid compound identification. While this method allows for rapid identification of compounds, there are still many false-positive results. These data were also applied to Cytoscape visualization to form pie charts for initial visual comparison of differences between origins. MarkerView 1.2.1 software [9] was utilized for differential compound screening. This software analyzes mass spectrometry data from multiple batches and combines the predicted significance and *p*-values to find differential compounds. Through cluster analysis [8], the changes in the relative content of a small number of differential compounds were used to reflect the quality differences in different regions. Meanwhile, we also focused on the antioxidant activity of SF samples between different origins. The widely used and representative DPPH and ABTS free radical scavenging assays were selected [11].

In this study, we explored the differences in the chemical compounds of SF among different regions in different provinces through a multivariate statistical analysis method using the UPLC-Q-ZENO-TOF-MS/MS technique. The results of the above analytical methods were synthesized to identify and evaluate the origins of the compounds of SF from multiple sources and batches. The results of the study can preliminarily elucidate the effects of different ecological changes on its material basis. It is of great significance to promote the basic research and quality standardization of SF.

## 2. Results

### 2.1. Optimization of Extraction Conditions

Three preparation conditions were investigated in this experiment. These included extraction solvents (pure water, 25%, 50%, 75%, and 100% methanol), ultrasonic extraction time (30, 60, 90, and 120 min), and the material–liquid ratio (1:10, 1:25, 1:50, and 1:100 *w*/*v*). The results showed that 75% methanol as a solvent, a solid–liquid ratio of 1:50, and an ultrasonic extraction time of 60 min were the optimal conditions. The liquid-phase response was more intense, and more substances could be detected under these conditions. Therefore, 75% methanol, a 1:50 solid–liquid ratio, and a 60 min ultrasonic extraction time were selected as the optimal extraction conditions. The figure results of the experimentally optimized conditions are shown in Supplementary Material S1.

### 2.2. Optimization of UPLC-Q-ZENO-TOF-MS/MS Conditions

In this study, the effects of water–methanol, methanol–0.1% (*v*/*v*) formic acid/water, acetonitrile–water, and acetonitrile–0.1% (*v*/*v*) formic acid/water as mobile phases on the separation of sample peaks were investigated. It was found that acetonitrile–0.1% (*v*/*v*) formic acid/water was the mobile phase with the best separation effect. In addition, by comparing the positive- and negative-ion flow diagrams of mass spectrometry, it was found that the sample had fewer peaks and a lower response in negative-ion mode. Therefore, the cation mode was finally selected for sample information collection. The results of the experiment are shown in Supplementary Material S1.

### 2.3. Identification of the Compounds of SF

**Figure 1:**
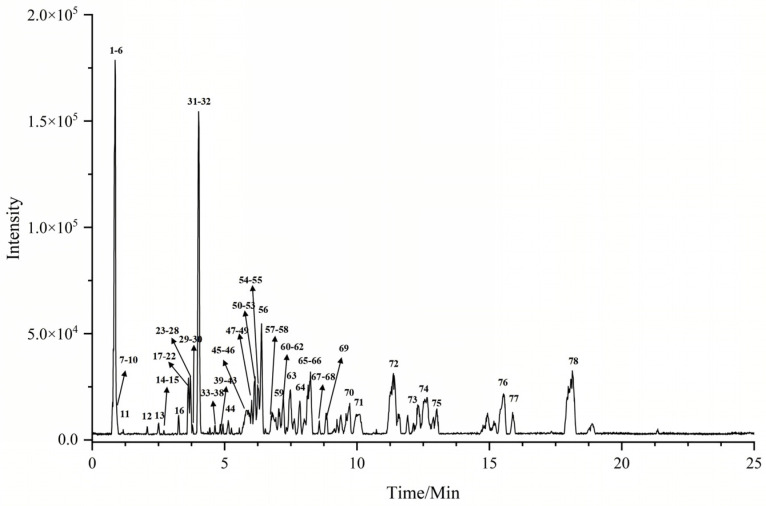
The base peak chromatogram (BPC) of SF in positive-ion mode.

The experiments were carried out according to the determined chromatographic and mass spectrometric conditions. The results were analyzed and chemically characterized using 60 batches of SF mass spectrometry samples from 20 origins in 3 provinces. At the same time, these mass spectrometry data were analyzed using molecular network node enrichment. The deduced molecular formulae were imported into PeakView again for validation to remove false-positive compound results. The results showed that the chemical compounds identified in the aquatic plants from Bobai County, Yulin, Guangxi, were more comprehensive, and the relative responses were higher. As shown in Figure 1, the base peak chromatogram (BPC) of groundwater from Bobai County, Yulin, Guangxi, was shown in positive-ion mode. A total of 78 compounds were identified, including 18 steroids, 16 steroidal saponins, 14 triterpenoid saponins, 7 flavonoid glycosides, 23 alkaloidal amino acids, and other chemical compounds. The details of the identified compounds are shown in Table 1. Their corresponding structures and CAS numbers are presented in Supplementary Material S2.

#### 2.3.1. Identification of Phytosterols and Their Glycosides

Phytosterols are a class of organic compounds containing a cyclopentane-parallel polyhydrophenanthrene structure. There are different alkane side chains attached to its C_17_ position. This class of compounds is an important metabolite in plants and exists mostly in free form or conjugated form, and it is an important compound of the membrane structure of plant cells. A total of 34 steroids were identified in this study, including 16 steroidal saponins, 9 phytosterols, and 9 spirostanes, as well as other steroidal compounds.

The structural classification of steroidal compounds was used to summarize a total of four categories of steroidal compounds contained in SF. For example, compounds **28**, **30**, **31**, **32**, **35**, **41**, **46**, **48**, and **49** belong to phytosterols with the same parent nucleus, and their characteristic diagnostic ions are *m*/*z* 319, *m*/*z* 301, *m*/*z* 165, and *m*/*z* 157 fragment ions. Compounds **56**, **58**, **63**, and **72** have a spirosteroidal structure belonging to the second class. Compounds **36**, **43**, **45**, **47**, and **76** belong to the third group and are other steroidal compounds. The fourth category is phytosteroid saponins. The main ion fragmentation peaks were *m*/*z* 301, *m*/*z* 283, and glucosyl representative fragmentation ion *m*/*z* 179, which represented compounds **18**, **23**, **26**, **39**, **40**, **42**, **44**, **57**, and **65** with the same phytosterol parent nucleus, while the compounds **17**, **21**, **24**, **19**, **25**, **27**, and **52** were the other classes of phytosteroid saponins.

**Figure 2:**
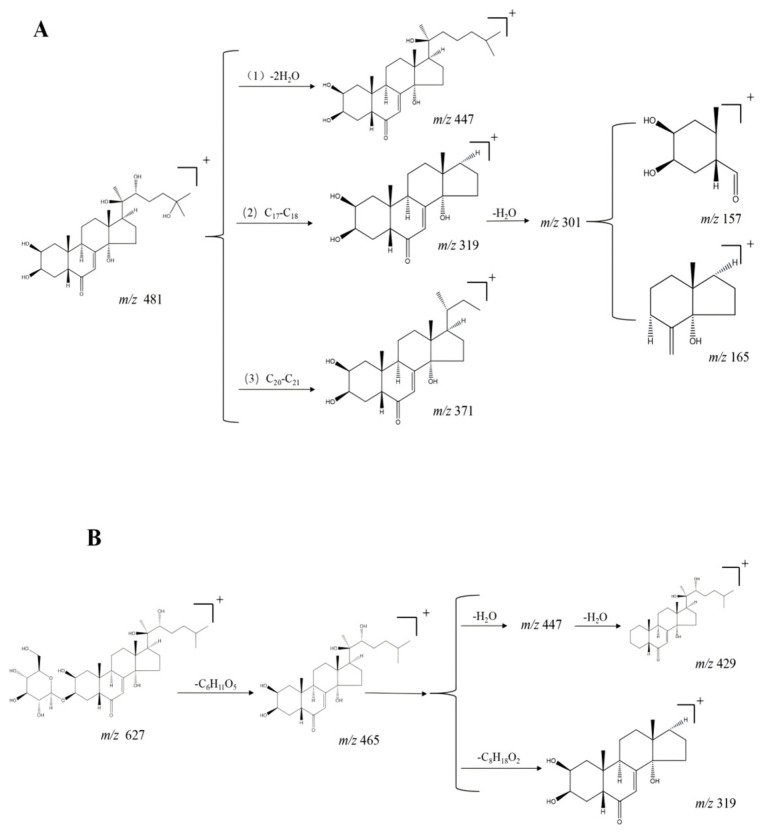
Schematic representation of steroid and steroidal saponin cleavage patterns in positive-ion mode. (**A**) Cleavage pattern of steroid compound **31**, hydroxyecdysone, and (**B**) cleavage pathway of steroidal saponin compound **42**, hydroxyecdysone-3-*O*-α-d-mannose—O.

Three main cleavage pathways of phytosterols were found by summarizing [44]. One, the mother nucleus ion of phytosterols, was only dehydrated and fractured, producing n hydroxyl fragments and forming [M + H - nH_2_O] ^+^ ion peak fragments. The second was the fracture of the C_17_–C_18_ bond in the branched chain of the sterol parent nucleus, which results in the formation of the characteristic ion fragmentation peaks of *m*/*z* 319 after the cleavage. In the last, a break in the C_20_–C_21_ bond in the aliphatic hydrocarbon chain of C_17_ of the sterol occurred. As compound **31** (hydroxyecdysone) has a similar cleavage pattern, its cleavage pathway is shown in Figure 2A. Phytosteroid saponin components are mainly composed of two parts: phytosterols and sugars. Their main cleavage modes are loss of sugar chains, side chains, and dehydration. For example, for compound **42**, its quasi-molecular ion peak is *m*/*z* 627 [M + H]^+^, the secondary fragment ions are *m*/*z* 465 [M + H-C_6_H_11_O_5_] ^+^, *m*/*z* 447 [M + H - C_6_H_13_O_6_]^+^, *m*/*z* 429 [M + H - C_6_H_15_O_7_]^+^, and *m*/*z* 319 [M + H - C_14_H_29_O_7_]^+^, and its cleavage pathway is shown in Figure 2B. Combined with the literature mass spectrometry data, it was confirmed as compound **42** with molecular formula C_33_H_54_O_11_, as hydroxyecdysone-3-*O*-α-d-mannose—O [13].

#### 2.3.2. Identification of the Steroidal Saponins

**Figure 3:**
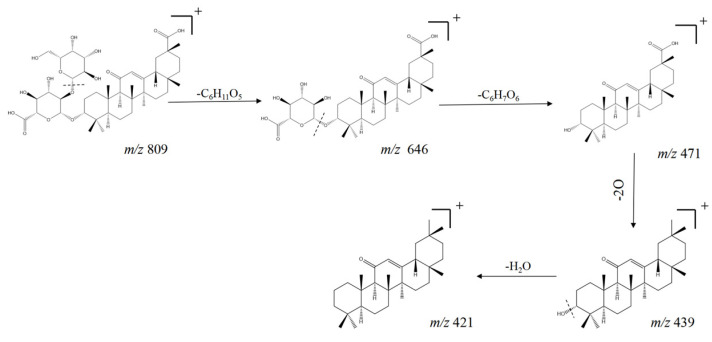
Cleavage pathway of compound **68** (QUDA-Glc-Glc) in positive-ion mode.

Due to the triterpenoids having more than one six-membered ring skeleton, their structure as a stable, six-membered ring bond was not easy to break. Therefore, triterpenoid saponins generally existed in the free state or combined with sugar to form glycosides or esters. Their glycosidic bond in the mass spectrum was often broken to obtain glucose fragments, such as glucose, rhamnose, or arabinose, while the hydroxyl group (-OH) and carboxyl group (-COOH) attached to the mother nucleus ring of the saponin were easily lost by cleavage. Compounds **53**, **55**, **59**, **60**, **61**, **66**, **68**, and **71** are oleanane-type triterpenoid saponins with the same parent nucleus, while compounds **14**, **33**, **50**, **51**, **54**, and **67** are other classes of triterpenoid saponins. The triterpenoid saponins shared the ionic peak fragments *m*/*z* 181 and *m*/*z* 163 of sugar group fragments. However, the oleanane-type triterpenoid saponins also shared ion peak fragments *m*/*z* 439 and *m*/*z* 163. In the case of the more responsive compound **68**, the loss of the two sugars first yielded the sugar ion fragmentations of *m*/*z* 163 and *m*/*z* 175, and the remaining fragmentation ions were *m*/*z* 457.0605 and *m*/*z* 439. At the same time, the carboxyl group at C_20_ position was easily lost to produce the O fragment, and its cleavage pattern is shown in Figure 3.

#### 2.3.3. Identification of Other Compounds

**Figure 4:**
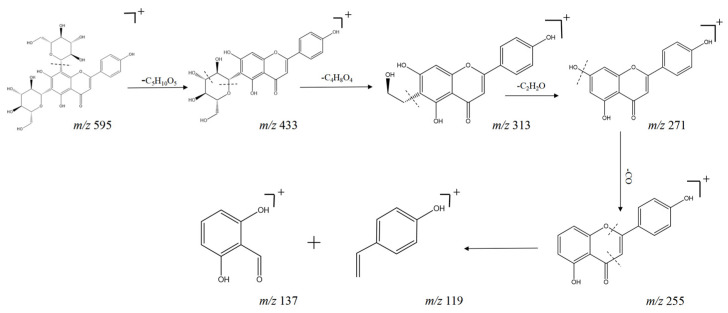
Possible cleavage pathways for compound **22** (Oroxin B) in positive-ion mode.

Flavonoid glycosides in SF were less and relatively low. The compounds were prone to glycosidic bond cleavage, neutral loss of glucose, rhamnose, or xylose, and other characteristic fragments, and their glycosidic structure could continue to lose CH_3_, CO_2_, or flavonoid glycoside C ring RDA cleavage. Compounds **15**, **16**, **22**, **29**, **34**, **38**, and **64** were detected with fragment ions *m*/*z* 271 and *m*/*z* 255. Further classification revealed that compounds **15**, **29**, and **64** are flavonol glycosides, and **16**, **22**, **34**, and **38** are flavonoid glycosides. The quasi-molecular ion peak of compound **22** was *m*/*z* 595 [M + H] ^+^. Firstly, the *m*/*z* 433 [M + H - Glc] ^+^ fragment ion was obtained by losing one molecule of glucose, and in losing one molecule of C_4_H_8_O_4_ or C_6_H_10_O_5_, it would obtain two fragment ions of *m*/*z* 313 and *m*/*z* 283. The *m*/*z* 255 was obtained by losing another molecule of CO from *m*/*z* 283. A further RDA cleavage reaction occurred, producing fragment ions of *m*/*z* 137 [C_7_H_4_O_3_] ^+^ and *m*/*z* 119 [C_8_H_6_O] ^+^, combined with the mass spectral cleavage pattern. The compound was identified to be Oroxin B, with molecular formula C_27_H_30_O_15_ [21]. The cleavage pattern is shown in Figure 4.

**Figure 5:**
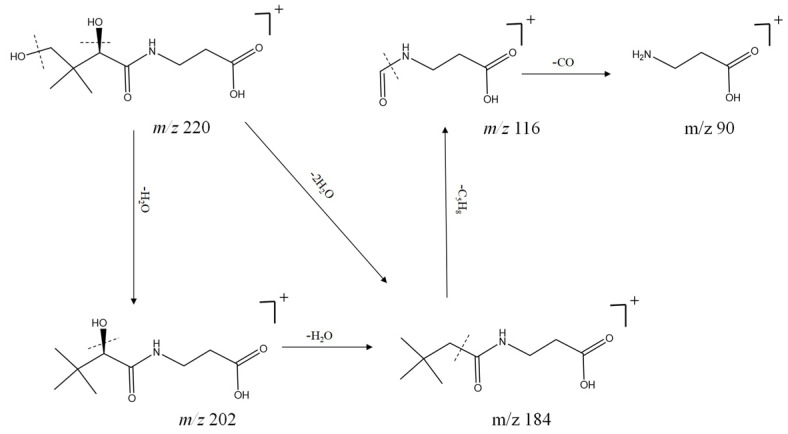
Positive-ion mode cleavage pathway of compound **10** (C_9_H_17_NO_5_).

In addition, it was analyzed and found that there were more nitrogenous organic compounds of amino acids and alkaloids in SF, which have complex chemical structures but have a wide range of biological activities. For example, compounds **1**, **4**, **6**, **8**, and **10** are amino acids, and compounds **2**, **9**, **11**, **12**, **20**, and **73** are alkaloids. These compounds changed in various ways during cleavage and were prone to loss of H_2_O and CO. Alkaloids were prone to α-cleavage and loss of linking substituents, which resulted in the fragment ion peak CO. For example, in compound **10**, with its quasi-molecular ion peak *m*/*z* 220 [M + H] ^+^, two hydroxyls were removed first to obtain the *m*/*z* 184 [M + H - 2H_2_O] ^+^ fragment ion peak, loss of one molecule C_5_H_8_ yielded *m*/*z* 116 [M + H - C_5_H_12_O_2_] ^+^, and other fragment ions *m*/*z* 90 and *m*/*z* 116. The cleavage pattern is shown in Figure 5.

### 2.4. Molecular Network Visualization and Analysis of SF Mass Spectrometry Data

**Figure 6:**
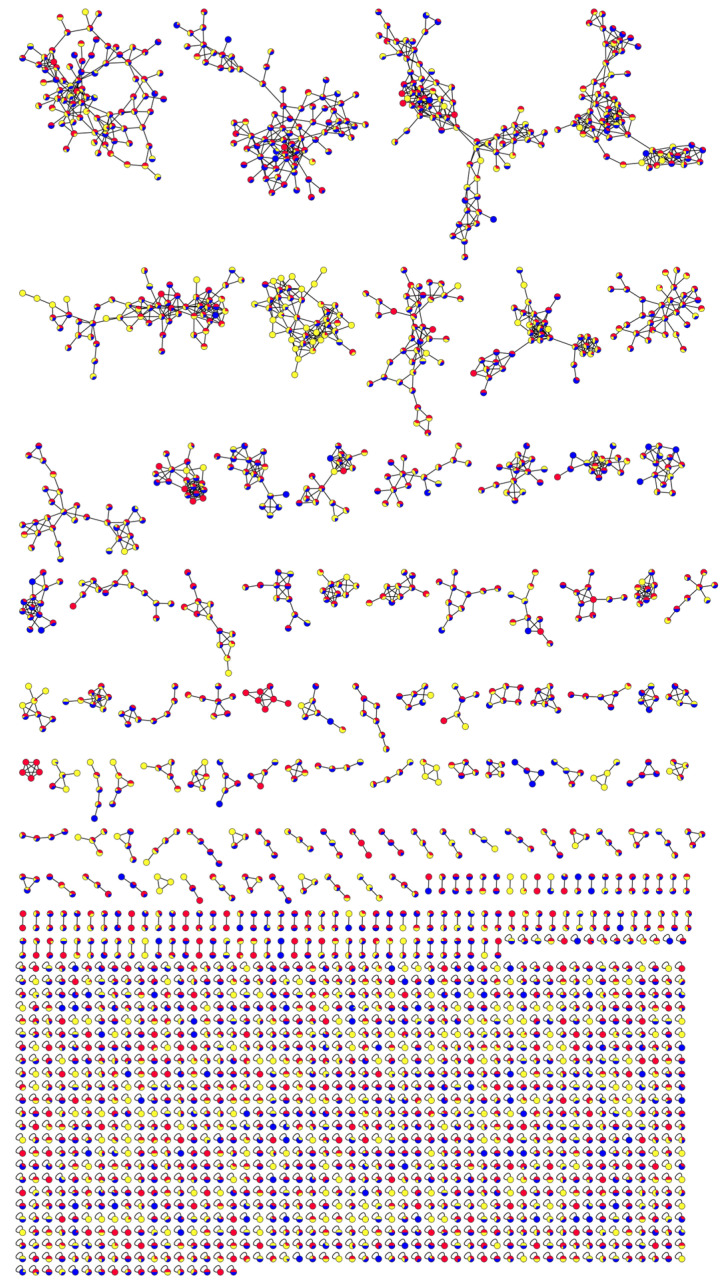
Visualization of the molecular network in the positive-ion mode of SF.

The molecular network analysis of the mass spectrometry information was identified and then visualized, and the results are shown in Figure 6. Based on the characteristic fragment ions of the compounds, a total of four classes of molecular network clusters of compounds were attributed. The content contained four major classes of steroids, saponins, flavonoids, and alkaloids. Based on the similarity of the GNPS molecular networks. Through fragments and mass spectrometry cleavage patterns were carried out to further characterize the linkages between the chemical compounds.

The first network consisted mainly of steroids and their saponins, characterized by the phytosterols of compounds **31**, **32**, and **46**. They were in the same network as some steroidal saponins, such as compounds **25** and **26**. This network allowed for further validation of the link between steroids and compounds such as steroidal saponins. The third network was dominated by triterpenoid saponins and contained compounds **33**, **51**, **61**, and **66**, as well as other compounds. These compounds have the same parent nucleus. Therefore, the third network was a network of triterpenoid saponin compounds aggregated from a class of compounds with oleanocarpane as the parent nucleus. In contrast, nitrogen-containing alkaloids, such as compounds **12** and **73** for fatty amido-enones and other compounds, were distributed in the eighth network, which was characterized by the presence of nitrogen in the compounds. Some compounds with flavonoids were clustered independently in the eleventh clustering network due to their structural similarity, containing compounds **16** and **22**. Comprehensive analysis of these chemical compound networks can be visualized regarding the classification of SF chemical compounds. A preliminary validation of the cleavage law of the partial chemical compounds of SF was carried out to correlate the related compounds, with the disadvantage that there were certain false positives. Therefore, it was necessary to analyze and summarize the differences in the chemical compounds of SF from different origins and sources by combining various methods.

### 2.5. Differential Analysis of the Chemical Compounds of SF from Different Origins

**Figure 7:**
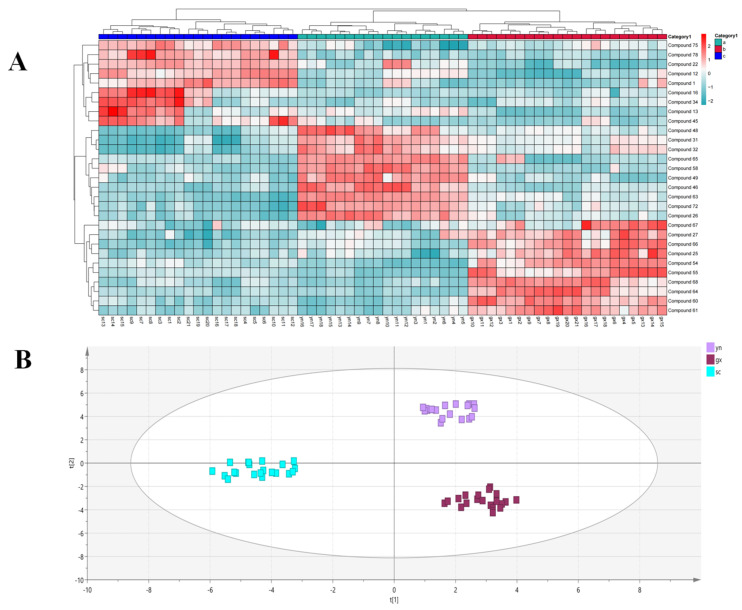
(**A**) Heat map of compounds differing in SF of different origins. (**B**) Results of principal compound analysis of SF samples among three provinces.

SF mass spectrometry data of 60 batches from 20 origins were imported into MarkerView. A *t*-test was performed to screen out compounds with *p* < 0.05, and a total of 29 compounds were identified. Clustered heat maps were plotted using the response value of each peak of the mass spectrometry data in each batch as its relative content. The visual description of the variability of multiple origins and batches was realized. Among the 29 compounds screened, they consisted of 13 steroids and their glycosides, 7 triterpenoid saponins, 4 flavonoid glycosides, and 5 other compounds. As shown in Figure 7A, the SF samples from Yunnan had higher relative amounts of compounds **31**, **32**, **46**, **48**, and **49**. The relative response values of compounds **55**, **60**, **64**, **66**, and **68** were significantly higher in the samples from Guangxi. The SF samples from Sichuan contained generally higher relative amounts of compounds **1**, **2**, **22**, **78**, and **75**. Further study of the climatic geography of these origins revealed that Guangxi region belongs to the subtropical monsoon climate, and due to the proximity of the ocean, the influence of the maritime climate is greater, and its steroid and saponin content is higher. Yunnan is in a variety of climates, such as the tropical monsoon climate and subtropical monsoon climate, with higher temperatures and higher contents of steroidal compounds. Sichuan, on the other hand, is at a higher altitude and under the influence of the plateau monsoon climate, and is rich in chemical compounds, with a relatively high content of alkaloids and flavonoids. This may be one of the reasons for the large differences in SF quality among the provinces.

Cluster analysis of the relative content of chemical compounds within each province revealed that Jinghong Xishuangbanna, Yuxi Xinping, and Kunming Yiliang from Yunnan were clustered into one group. The main reason was the high relative content of the three steroidal compounds, **31**, **32**, and **48**. Ninglang from Lijiang, Dongchuan from Kunming, and Xishan from Kunming were clustered into the second group. The SF samples from Yulin Bobai, Yulin Bobai, and Nanning Longan in Guangxi Province were clustered into one group because of the high relative contents of compounds **25**, **54**, **55**, and **67** from these three origins. In Guangxi, Yulin Beiliu, Yulin Yuzhou, Guilin Quanzhou, and Yulin Rongxian formed the second largest group because the relative contents of compounds **60**, **61**, **64**, and **68** were generally higher. The Sichuan provinces Kangding Ganzi Prefecture, Danba Ganzi Prefecture, Dachuan Dazhou, and Jinchuan Aba Prefecture were clustered into one group due to the low relative content of two flavonoid glycosides, compounds **16** and **34**. Huidong Liangshan, Liangshan Puge, and Meishan Dongpo from Sichuan were clustered into the second largest group, with higher responses for compounds **13**, **16**, **34**, and **45**. The results showed that the relative contents of steroidal compounds were higher in Yunnan Province, while the contents of steroidal saponins and triterpenoid saponins were significantly higher in Guangxi. However, the relative contents of flavonoids and other compounds, such as alkaloids, were higher in Sichuan. As the plant’s growing environment became harsher, at the same time, the relative content of steroidal compounds and saponins of the genus decreased, and its chemical compounds became richer. Therefore, the relative content of compounds can be used to distinguish herbs of different origins. This also reflects that the quality of herbs from different origins varies greatly. Although the quality of SF from Guangxi Province was relatively stable and rich in compounds, differences in compounds and their relative contents can lead to unstable clinical effects. This study provides ideas to stabilize the quality of SF.

The 60 batches of SF samples contained shared constituent compounds **31** and **32**, which have been reported in the literature [5,6] to possess anti-inflammatory, analgesic, and neuroprotective effects. This suggests that these compounds may play an important role as pharmacodynamic compounds. Although these two compounds are common, they may be the key compounds affecting the quality of the drug due to their different relative contents. However, the study of the differential compounds showed that there are still many compounds that are in the preliminary stage of research, such as compounds **54** and **61**, which are triterpenoid saponin compounds with high relative content, whose pharmacological studies are still unclear. For the relatively high content of compounds **66** and **68** in Guangxi, their antitumor and hypoglycemic effects have been reported in the literature [13,45]. These compounds may reflect the pharmacological activities of SF to some extent.

### 2.6. Results of SF Samples of Different Origins in DPPH and ABTS Radical Scavenging Assays

**Figure 8:**
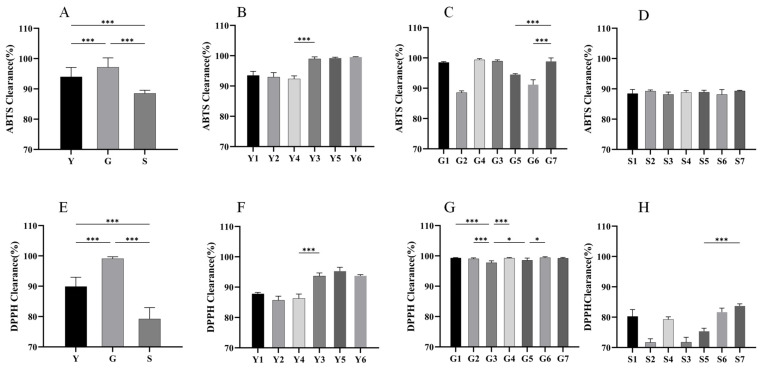
Antioxidant activity of ABTS (**top**) and DPPH (**bottom**) of SF. Notes: (**A**,**E**) ($\bar{x}$ ± s, Y, n = 18, G or S, n = 21) and (**B**–**D**,**F**–**H**) ($\bar{x}$ ± s, n = 3). * *p* < 0.05 and *** *p* < 0.001. Y, Yunnan; G, Guangxi; S, Sichuan. Numbers represent the origin (see File S2 of the Supplementary Materials for origin information). Clearance rate (%) = [(A control − A sample)/A control] × 100%.

Yuan L. [46] found that the antioxidant activity capacity of *Psammosilene tunicoides* W.C.Wu et C.Y.Wu was related to the relative content of steroids and their saponins with different origin sources. In this study, the differences in chemical compounds and their relative contents of SF samples collected from several origins were compared. The quality differences in SF from different origins were further verified by the differences in their antioxidant activities. The 60 batches of SF were firstly divided into 3 major groups according to provinces, as shown in Figure 8A,E, and then each group was further divided into different subgroups by origin information, as shown in Figure 8B–D,F–H. The results showed that these SF samples possessed significant antioxidant activities in both ABTS and DPPH radical scavenging assays. Among them, SF from Guangxi showed the most significant antioxidant capacity in both ABTS and DPPH antioxidant assays, and the range of antioxidant activity was wider than that of W from the other two provinces. Although the SF samples from Sichuan Province also possessed stronger antioxidant activities, they still fell short of the samples from Guangxi and Yunnan.

We also compared the antioxidant capacity of SF from different regions within each province. As shown in Figure 8B,F, the SF antioxidant capacity of Yuxi Xinping, Kunming Yiliang, and Jinghong Xishuangbanna in Yunnan Province was stronger than that of the SF samples from the other three origin sources. Figure 8C,G shows that the SF samples from Guangxi Province had stronger scavenging capacity for DPPH and slightly different antioxidant capacity for ABTS. For example, the scavenging ability of origins Yulin Bobai and Nanning Long’an were weaker for ABTS. Figure 8D,H shows that there was no significant difference in the ABTS scavenging capacity of SF from Sichuan Province, while three provinces, Liangshan Puge, Meishan Dongpo, and Huidong Liangshan, had significantly weaker DPPH scavenging capacity than the other four provinces.

In the three provinces, those from Yunnan origin had higher antioxidant capacity and their relative phytosterol compound was also higher. The SF samples from Guangxi Province had more significant antioxidant activity, which was related to its sterol content, along with possessing a higher relative content of saponins. On the other hand, the SF sample from Sichuan Province had the weakest antioxidant activity due to the high relative content of alkaloids and flavonoids in its compounds, which again verified that its antioxidant activity was related to the relative content of sterols and saponins. Although these SF plant samples contained many common compounds, there were significant differences in their antioxidant activities. In this study, the differences in relative compound content and antioxidant capacity were analyzed in combination. The antioxidant activity was found to be related to the higher abundance of steroids, such as ecdysterols and triterpene saponins; thus, the saponin sterols may be responsible for the more significant antioxidant activity of SF.

## 3. Discussion

The results of the study showed that the different environments in which the plants were grown made the relative contents of the constituent species in SF plants vary considerably. Therefore, standardization for the quality of SF herbs was difficult. The present study provided a better method to distinguish SF from different origins and, at the same time, to assess their quality. In this study, SF samples from three provinces (Guangxi, Yunnan, and Sichuan) were systematically analyzed. The origin was categorized by establishing a link between different compounds and differences in antioxidant capacity. These results provided fundamental data for the study of SF metabolites, the in vivo synthesis mechanism of related components, and the assessment of the quality of the herb. Due to the relative abundance and high content of steroidal and saponin components in the herb, their high molecular weight made identification difficult. Therefore, in the course of subsequent research, we will combine more accurate and extensive compositional identification techniques, such as DNA molecular identification technology, gene transcriptomics, and near-infrared spectroscopy, to analyze the quality of herbs at multiple levels and dimensions. This will provide a more comprehensive theoretical basis for the standardization of the quality of Chinese herbs. The shortcomings of this experiment were as follows: Firstly, there were many duplications of chemical compounds among the origins, and it was difficult to be precise about the extent to which a certain compound played a key role in the quality of SF. Secondly, there were difficulties in identifying compounds with large molecular weights, and there were no pharmacological studies on the compounds identified by acetonitrile.

## 4. Materials and Methods

### 4.1. Chemicals and Reagents

Methanol and acetonitrile of HPLC grade were obtained from Merck (Darmstadt, Germany), formic acid was purchased from Fischer Technologies (Czech Republic Lot: 220622), and deionized water was purchased from Watson (Lot: 202400123, Guangdong, Guangzhou, China). K_2_S_2_O_8_ (Lot: 20221220), ABTS (Lot: B2308190), and DPPH (Lot: M27HS179342) were purchased from Shanghai yuanye Bio-Technology Co., Ltd. (Shanghai China)

### 4.2. Collection and Preparation of Medicinal Herbs

A total of 60 batches of WaCao samples from 20 origins were collected from Yunnan, Guangxi, and Sichuan provinces through various channels. The samples were identified as dried roots of SF plant by Prof. Liu Xunhong (China), Faculty of Pharmacy, Nanjing University of Chinese Medicine (Nanjing, China). The plant samples were kept at Jiangsu Province Engineering Research Center of Classical Prescription, Nanjing University of Chinese Medicine. The source information of SF samples is shown in File S2 of the Supplementary Materials S3.

All the samples collected were pulverized into powder and passed through sieve No. 3 of the Chinese Pharmacopoeia, respectively. About 1 g of powder was weighed separately and accurately into a conical flask, then 50 mL of 75% (*v*/*v*) methanol in water was added accurately and weighed with a stopper. After sonication for 30 min (300 W, 40 KHZ), the samples were cooled to room temperature and 75% methanol was added to compensate for weight loss. The sample solution was filtered through filter paper and set at -4°C. Then, 2 mL of the extract was centrifuged at 13,000 rpm for 10 min and the supernatant was taken. The supernatant was filtered through a 0.22 µm membrane. At the same time, aliquots of 60 batches of SF sample solution were mixed and used to prepare quality control samples. Simultaneously, samples were analyzed every 5 samples to ensure instrument stability.

### 4.3. UPLC-MS/MS Conditions Analysis

The detection was performed using a UPLC-ZENO-Q-TOF-MS/MS 7600 liquid-mass spectrometer with an autosampler (AB SCIEX, Framingham, MA, USA), Analystl.6 chromatographic workstation, and mass spectrometry analysis software, such as PeakView 1.2. The chromatographic column was the Agilent polaris 3 C18 A (2.1 × 100 mm, 1.8 µm). The gradient elution was performed with 0.2% (*v*/*v*) formic acid–water as mobile phase A and acetonitrile as mobile phase B: 0~4 min (10~30% B); 4~15 min (30~51% B); 15~20 min (51~51% B); 20~22 min (52~90% B); 22~23 min (90~90% B); 23~25 min (90~10% B). Flow rate: 0.3 mL/min, injection volume: 3 µL, column temperature: 35 °C, atomizing gas flow (GS1) and auxiliary gas flow (GS2): 55 psi, ion spray floating voltage (ISVF): 5500 V, collision energy (CE): 35 eV, and de-embodied potential: 100 eV. The data acquisition range for each sample was 50–1500 Da. The sample collection time was 25 min.

### 4.4. Molecular Network and Compound Identification Analysis

The original SF mass spectrometry files were converted to mzML format. The above files, which were uploaded to the GNPS website (https://gnps.ucsd.edu/, accessed on 12 July 2024) via WinSCP software 6.1, were used to form a molecular network. The resulting molecular network node information and clusters were visualized and post-processed by Cytoscape 3.8.0 software, and the network was analyzed and identified for associated compounds. The molecular formula results obtained from the analysis were used to exclude false positives by PeakView, and the ion fragmentation information of the MS/MS of the remaining compounds was compared with CNKI (https://kns.cnki.net/, accessed on 22 July 2024), SciFinder (https://scifinder.cas.org/, accessed on 23 July 2024), PubChem(https://pubchem.ncbi.nlm.nih.gov/, accessed on 23 July 2024), and related literature. Information from PeakView 1.2 and the GNPS molecular network was utilized to mutually validate the analysis.

### 4.5. Comparative Analysis of the Chemical Compounds and Antioxidant Activity of SF from Different Origins

The raw mass spectrum file of the SF sample was imported into MarkerView 1.2.1(Sciex AB, Framinghan, MA, USA), the retention time was set to a minimum of 0.5 min and a maximum of 25 min, and the rest of the parameters were set to default values. The noise threshold was 100, and isotope ions were removed. Sorting by origin and starting to process multiple batches, the raw mass spectral data were subjected to peak matching, peak alignment, and noise filtering, and normalized to analyze the relative content of shared peak compounds. The data were analyzed by peak matching, isotope ion removal, and shared peak analysis. Finally, the *t*-test was performed to screen out *p*-values of (*p* < 0.05) regarding the molecular weight of compounds. The identified compounds were analyzed as SF differential compounds of different origins. The response values of the identified differential compounds were derived and plotted as heat maps for visual comparison of the relative contents.

The SF samples were subjected to DPPH and ABTS free radical scavenging assays [46], and their antioxidant activities were comprehensively analyzed and evaluated. Clearance units of ABTS and DPPH antioxidant assays are commonly expressed as a percentage. The samples of different concentrations of SF (Samples preparation is described in Section 2.1) were subjected to preliminary screening experiments for DPPH vs. ABTS sample concentrations. The samples were initially screened at concentrations of 100 mg/L, 40 mg/L, 20 mg/L, and 10 mg/L. The ABTS clearance was 99.82%, 91.34%, 88.23%, and 49.23%. The initial clearance rates of the DPPH assay were 99.17%, 89.13%, 77.14%, and 36.14%. The scavenging rates calculated based on the above equations were processed using GraphPad Prism 9.5.0 software. The IC_50_ values of the samples were 10.16 mg/L and 12.40 mg/L for the removal of DPPH and ABTS radicals. The DPPH and ABTS free radical scavenging assays were performed according to literature methodology. Finally, the experiments were carried out in terms of the 40 mg/L raw drug concentration. The differences in SF compounds and contents and their antioxidant activities from different origins were comprehensively analyzed.

## 5. Conclusions

In this study, the chemical compounds of SF from different origins in three provinces were analyzed by UPLC-Q-ZENO-TOF-MS/MS. Based on the relevant mass spectrometry data and literature, a total of 78 chemical compounds were identified. The cleavage pathways of triterpenoid saponins, steroidal saponins, steroids, and flavonoids were preliminarily summarized and speculated. The chemical compounds of SF from different origins were identified by the *t*-test and other methods, and the relative contents of some steroidal and saponin compounds were compared. Finally, we preliminarily confirmed that the quality of SF from Guangxi Province was better. These findings helped us to better understand the chemical compounds of SF from different origins and their quality differences and provide data for further exploration of the material basis of SF and pharmacological studies.

## Figures and Tables

**Table 1:** Identification of 78 compounds in SF.

No.	t_R_ min	Molecular Formula	[M + H] ^+^	Error (ppm)	MS^2^	Compound	Ref.
1	0.78	C_6_H_14_N_4_O_2_	175.1191	0.6	116.0728, 72.0807, 70.0722, 60.0600	Arginine ^a^	[12]
2	0.87	C_11_H_12_N_2_O_2_	205.0964	−3.6	205.0964, 159.0712, 118.0545, 74.0251	L-5-Hydroxytryptophan ^a^	[13]
3	0.89	C_14_H_10_N_2_O_4_	271.0804	3.5	271.0806, 109.0291	Siliendine A ^a^	[14]
4	0.89	C_5_H_9_NO_2_	116.0705	−0.8	116.0709, 70.0663	Proline ^a^	[12]
5	0.89	C_29_H_44_O_5_	473.3252	−2	445.3173, 427.3191, 161.0903	3β-Acetoxy-(25R)-5α-spirostan-12-one ^a^	[15]
6	0.92	C_9_H_11_NO_3_	182.0813	0.5	182.0817, 119.0497, 91.0569	Tyrosine ^a^	[12]
7	1.15	C_9_H_6_O_2_	147.0438	−1.6	147.0438	Coumarin ^a^	[16]
8	1.16	C_6_H_13_NO_2_	132.1019	−0.3	86.0959, 69.0699, 56.0490	Isoleucine ^a^	[12]
9	1.32	C_12_H_12_N_2_O_2_	217.0971	−0.5	144.0826, 127.0541, 171.0928	1,2,3,4-Tetrahydro-1H-pyrido[3,4-b] indole-3-carboxylic Acid ^a^	[13]
10	1.43	C_9_H_17_NO_5_	220.1179	−0.4	205.1581, 90.0546, 72.0443	Pantothenic acid ^b^	[17]
11	1.82	C_17_H_22_N_2_O_7_	367.1502	0.5	332.1155, 229.0973, 188.0705, 146.0600	N-(1-Deoxy-1-fructosyl) Tryptophan ^b^	[17]
12	2.01	C_11_H_9_NO_2_	188.0706	0.1	146.0604, 118.0653, 91.0539	Indoleacrylic acid ^b^	[18]
13	2.46	C_18_H_24_O_12_	433.1337	−0.8	145.0493, 127.0399, 85.0283	Licoagroside B ^b^	[19]
14	3.06	C_45_H_72_O_20_	933.4657	−3.5	933.4667	Macrostemonoside I ^b^	[20]
15	3.13	C_27_H_30_O_16_	611.1591	−2.6	611.0689, 383.0768, 329.0637, 299.0539	Vincetoxicoside A ^a,b^	[21]
16	3.24	C_26_H_28_O_14_	565.1547	−0.8	427.1007, 271.0812, 349.0703, 433.0598	Apiin ^b^	[21]
17	3.24	C_39_H_62_O_16_	787.4101	−1.2	625.3588, 463.3044, 427.2846, 301.1797	Pregn-5-en-3β,20(S)-diol-3-*O*-bis-β-d-glucopyranosyl-(l-2,1-6)-β-*D*-glucopyranoside ^a, b^	[13]
18	3.27	C_33_H_54_O_13_	659.3623	−2.2	659.3643, 443.2792, 425.2693, 407.2540	Tupistroside K ^b^	[22]
19	3.29	C_37_H_50_O_8_	623.3534	2.6	623.3372, 301.3236	Ecdysterone 2,3-Monoacetonide 22-*O*-Benzoate ^a, b^	[15]
20	3.59	C_18_H_18_N_3_O_5_	357.1172	−0.9	357.1169	Siliendine C ^a^	[14]
21	3.61	C_39_H_64_O_17_	805.4046	−1.7	465.3208, 301.3105, 426.2977, 363.2268	Sileneoside G ^a^	[23]
22	3.62	C_27_H_30_O_15_	595.1654	−0.5	433.1140, 283.0929, 313.0713, 271.0604	Oroxin B ^b^	[21]
23	3.68	C_39_H_62_O_15_	771.4153	−1.2	593.3193, 411.2659, 162.2843	Ophiopogonin R ^b^	[24]
24	3.68	C_39_H_64_O_16_	789.4241	−3.4	609.3670, 447.3123, 429.2993, 355.2274	Tupistroside L ^a^	[22]
25	3.69	C_33_H_52_O_11_	625.3575	−1.2	463.1238, 367.0820, 343.0815, 313.0715, 301.1798	Hydroxyecdysone-3-*O*-α-d-mannose—H_2_O ^a, b^	[13]
26	3.7	C_33_H_54_O_12_	643.3683	−0.8	481.3165, 445.2971, 371.2220, 165.1276, 127.2848	Tupistroside J ^a, b^	[13]
27	3.7	C_33_H_50_O_10_	607.3472	−0.8	607.3473, 427.2853, 283.1798	Caucasicoside A ^b^	[10]
28	3.71	C_19_H_26_O_5_	335.1847	−1.8	335.1847	Dehydro-8-gingerdione ^b^	[25]
29	3.73	C_28_H_44_O_7_	493.3152	−1.4	457.2950, 173.1342, 311.2003	Polyporoid B ^b^	[13]
30	3.75	C_27_H_44_O_9_	513.3045	−2.7	513.2899, 423.2436, 369.2074, 211.1110	26-Hydroxypolipodine B ^a^	[26]
31	4.01	C_27_H_44_O_7_	481.3157	−0.6	463.3076, 371.2248, 301.1801, 165.1292	Hydroxyecdysone ^a, b^	[13]
32	4.01	C_27_H_42_O_6_	463.3050	−0.8	445.2979, 301.1840, 283.1702, 81.071	3-Dehydroecdysone ^a, b^	[13]
33	4.01	C_42_H_64_O_17_	841.5618	−1.4	841.3145, 501.1545, 163.0599	Armeroside B ^a^	[27]
34	4.04	C_21_H_20_O_10_	433.1131	0.4	431.0699, 313.0908, 271.0695, 283.0608	Apigenin-7-*O* glucoside ^b^	[21]
35	4.05	C_27_H_44_O_8_	497.3107	−0.4	497.3115, 443.2790, 425.2714, 81.0699	Abutasterone^a^	[13]
36	4.05	C_34_H_52_O_9_	605.3545	−5.8	447.3715, 383.2166	Ecdysteroid ^a^	[28]
37	4.06	C_34_H_48_O_7_	569.3501	4.9	514.3484, 311.2843, 303.1743	2-Dehydroxyecdysterone-3-*O*-benzoate ^a^	[29]
38	4.11	C_22_H_22_O_11_	463.1233	−0.5	367.0800, 343.0796, 283.0243, 301.1794	Chrysoeriol-7-*O*-glucoside ^b^	[17]
39	4.11	C_33_H_52_O_10_	609.3626	−1.2	447.3109, 429.3000, 411.2892	20,22-Trihydroxy-6-Oxocholesta-7,14-dien-3-yl beta-d-glucopyranoside ^a^	[13]
40	4.66	C_35_H_58_O_12_	671.3982	−2.9	671.3982, 507.3372, 313.1837	Hydroxyecdysone-3-*O*-α-d-mannose + CH_2_CH_3_^a^	[13]
41	4.69	C_28_H_46_O_7_	495.3306	−2.2	459.3092, 357.2051, 237.1633, 131.0859	Makisterone A ^a^	[13]
42	4.83	C_33_H_54_O_11_	627.3732	−1.1	465.3192, 445.3121, 429.3010, 411.2906	Hydroxyecdysone-3-*O*-α-d-mannose—O ^a, b^	[13]
43	4.97	C_24_H_32_O_6_	417.2267	−1.1	417.2312, 335.2024, 187.1114, 163.0717	Sidisterone ^a^	[26]
44	5.01	C_33_H_54_O_10_	611.3670	−2.3	413.3051, 593.2316, 287.2004	2-Deoxyecdysone 22β-d-glycoside ^a^	[26]
45	5.13	C_27_H_42_O_3_	415.3202	−1.1	415.3201, 397.3096, 271.2064, 285.1843	Diosgenin ^b^	[30]
46	5.19	C_29_H_46_O_8_	523.3262	−0.7	445.2928, 427.2849, 165.1266, 110.7474	Viticosterone E ^a^	[26]
47	5.2	C_27_H_40_O_7_	477.2842	−0.9	477.2765, 423.2492, 459.2448, 223.0979	Lucidenic acid C ^b^	[31]
48	5.26	C_27_H_44_O_6_	465.3201	−2	429.3006, 411.2875, 233.1544, 159.0815	Ponasterone A ^a^	[13]
49	5.57	C_29_H_48_O_7_	509.3467	−1.1	473.3248, 371.2201, 223.1479, 455.1563	Makisterone C ^a, b^	[32]
50	5.74	C_56_H_88_O_26_	1178.5041	−1.9	805.3985, 499.3032, 453.3121, 163.0604,	Silenegallisaponin B ^a^	[33]
51	5.78	C_45_H_72_O_17_	884.4549	−3.8	749.4031, 426.3206, 327.3086, 145.1844	Sileneoside B-diacetonide ^a, b^	[34]
52	5.83	C_33_H_49_O_9_	590.3378	4.8	479.3005, 383.2894, 172.1786	a-Ecdysone 2,3,25-Triacetate ^a, b^	[35]
53	5.99	C_41_H_62_O_16_	811.4085	−3.1	473.3268, 325.1130, 163.0600, 145.0495	QUDA-GlcA-Ara/Xyl ^a, b^	[13]
54	6.04	C_54_H_86_O_26_	1147.5151	−1.4	517.3145, 469.1545, 163.0599	Armeroside D ^a, b^	[27]
55	6.04	C_48_H_72_O_21_	985.4619	−2	679.3699, 517.3156, 215.1379	Licorice saponin A_3_^a, b^	[19]
56	6.24	C_30_H_48_O_7_	521.3457	−3	457.3277, 207.0323, 187.1468, 189.1354, 143.0806	Ecdysterone 20,22-monoacetonide ^a^	[28]
57	6.29	C_39_H_60_O_16_	785.3945	−1.2	447.3103, 429.2993, 411.2878, 393.2778	Dracaenoside C ^b^	[36]
58	6.56	C_30_H_46_O_6_	503.3360	−1.5	485.3254, 467.3153, 301.1629, 187.1473	3β-16β-Dihydroxy-olean-12-ene-23,28-dioic acid ^a^	[13]
59	6.66	C_41_H_52_O_9_	793.3977	−3.6	426.3977, 311.3153, 301.1629, 187.1473	Ecdysterone 22,25-Di-O-benzoate ^a, b^	[37]
60	7.48	C_48_H_76_O_21_	989.4934	−1.8	503.3363, 485.3266, 457.3309, 439.3201 163.0595	Sinocrassuloside I ^a, b^	[13]
61	7.48	C_42_H_66_O_16_	827.4408	−1.9	629.3245, 503.3298, 439.3204, 163.1430	Dianchinenoside D ^b^	[38]
62	7.48	C_15_H_24_	205.1947	−2.1	205.085	α-Humulene ^a^	[21]
63	7.49	C_34_H_48_O_6_	553.3253	−1.8	445.3001, 427.2864, 313.2248, 165.1292	Tomentesterone B ^a^	[39]
64	8.03	C_27_H_42_O_7_	479.1813	0.5	479.3003, 317.1755, 443.2789	4-Dehydroecdysterone ^a^	[13]
65	8.19	C_39_H_60_O_14_	753.4034	−2.9	426.3202, 591.3079, 162.1846	Kingianoside A ^b^	[40]
66	8.23	C_54_H_84_O_25_	1133.5372	−0.2	307.1028, 289.0912, 163.0595, 145.0486	Tunicosaponin J ^a, b^	[13]
67	8.25	C_48_H_74_O_20_	971.4828	−1.8	439.3204, 307.1017, 163.0603, 145.0495	QUDA-(Glc)-(Glc-Glc) ^a, b^	[13]
68	8.25	C_42_H_64_O_15_	809.4302	−2	457.3298, 439.3200, 163.0596, 145.0493	QUDA-Glc-Glc ^a, b^	[13]
69	8.48	C_51_H_58_O_10_N_8_	943.4332	−1.8	943.4354, 332.1593, 302.1485, 261.1226	Silenin C ^a, b^	[3]
70	9.14	C_36_H_50_O_9_	627.3514	−2.1	426.4773, 301.3539, 363.2021, 162.2333	Viticosterone E 22-*O*-benzoate ^a, b^	[35]
71	9.68	C_69_H_100_O_30_	1409.6319	−3.8	485.3254, 955.4653, 775.2523	Sinocrassuloside X ^a, b^	[13]
72	11.39	C_30_H_46_O_5_	487.3408	−2.1	451.3190, 201.1622, 187.1464	Quillaic acid ^b^	[41]
73	11.94	C_48_H_74_O_21_	987.5050	−1.1	987.4961, 163.4233	Ameroside F ^a, b^	[27]
74	12.28	C_17_H_15_N_3_O_5_	342.0969	−3.6	342.1383	Siliendine D ^a^	[14]
75	12.66	C_18_H_26_O_12_	435.1488	−2.1	435.0381, 127.0282, 92.0333	Canthoside C ^b^	[42]
76	15.91	C_48_H_74_O_22_	1003.4725	−1.9	1003.4725	Armeroside C ^a^	[27]
77	16.44	C_48_H_66_O_8_N_8_	883.5061	−1.7	883.5079, 211.1427, 70.0646	Silenin B ^a^	[3]
78	18.11	C_10_H_8_O_2_	161.0596	−0.7	133.0644, 120.0406, 92.0383	2-Methylchromone ^b^	[43]

^a^: Compounds identified through the literature and the PubChem website. ^b^: Compounds identified through the GNPS website.

## Data Availability

The data presented in this study are available within the article.

## Supplementary Materials

The following supporting information can be downloaded at: https://www.mdpi.com/article/10.3390/molecules29204817/s1. Supplementary Materials S1: The experimentally optimized conditions. Supplementary Materials S2: Compounds’ corresponding structures and CAS numbers. Supplementary Materials S3: Table S1: The source information of the SF samples.
