# Supplementary material for: Comprehensive Evaluation of Quality and Differences in Silene viscidula Franch from Different Origins Based on UPLC-ZENO-Q-TOF-MS/MS Compounds Analysis and Antioxidant Capacity
Source: Molecules. 2024 Oct 11;29(20):4817. doi: 10.3390/molecules29204817 (PMC11509892; doi:10.3390/molecules29204817)
Supplement: Supplementary file 1 [file molecules-29-04817-s001.zip › Supporting material S1 The experimentally optimized conditions.pdf]

In order to better detect and identify the compounds, we optimized the mobile phase, extraction solvent, ultrasonic extraction time and feed-liquid ratio for Silene viscidula Franch after mapping the UPLC conditions. After comparison, we chose 75% methanol as the extraction solvent, the sonication time was 30 min, the extraction feed-liquid ratio was 1:50, and the mobile phase was acetonitrile and (0.1%) water. The results of mass spectrometry data showed that the conditions were suitable for the separation and identification of the samples, The following are some of the results of the inspection process

Examination of different mobile phases for UPLC

Acetonitrile-water

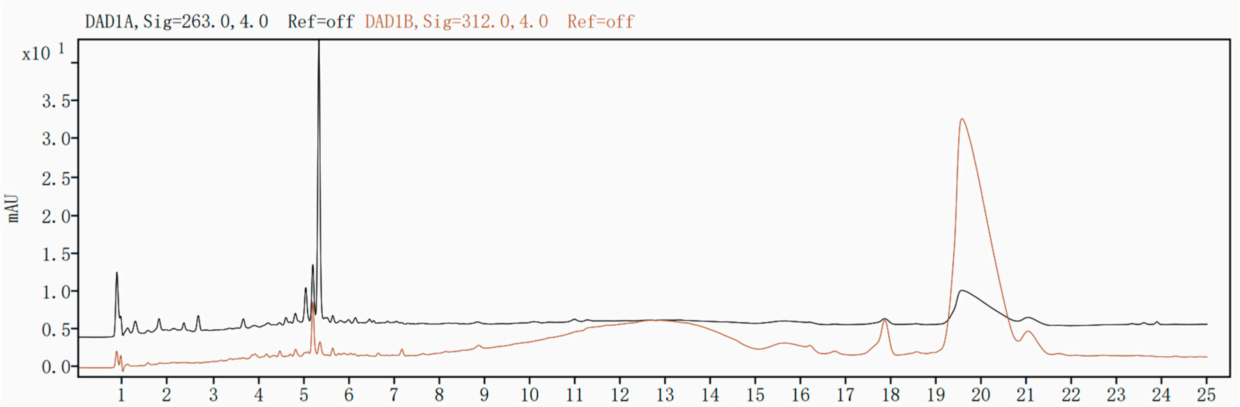

t/min

Methanol-water

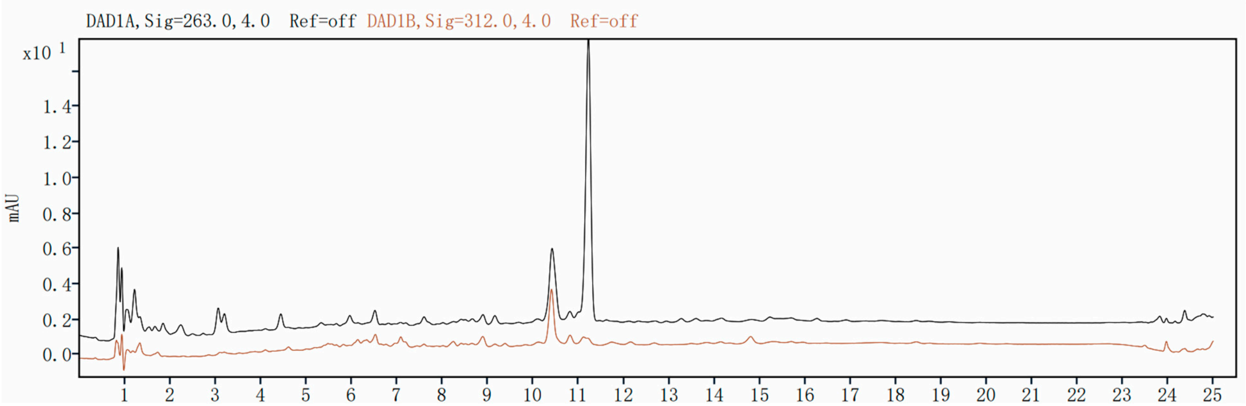

t/min

Methanol-0.1% (v/v) formic acid/water

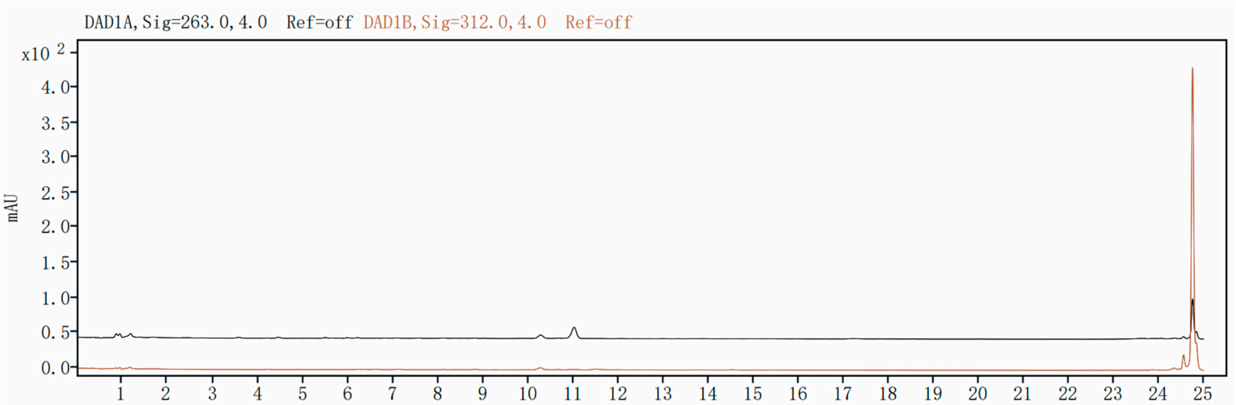

t/min

Acetonitrile-0.1% (v/v) formic acid/water

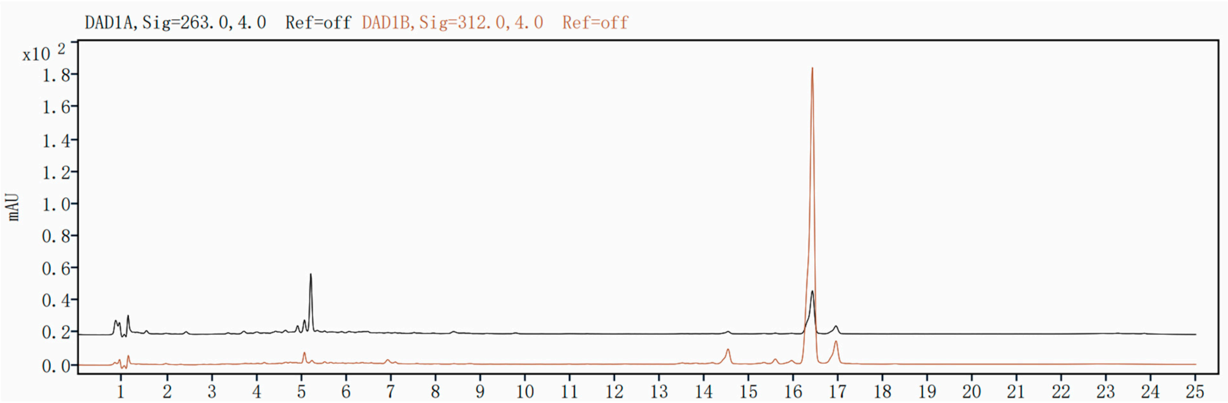

t/min

Extraction Solvents

Water

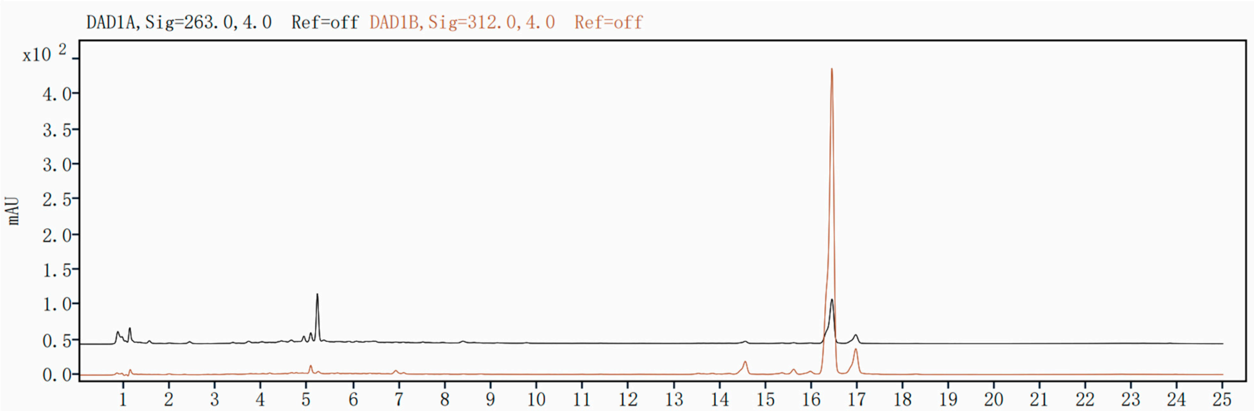

t/min

25% methanol

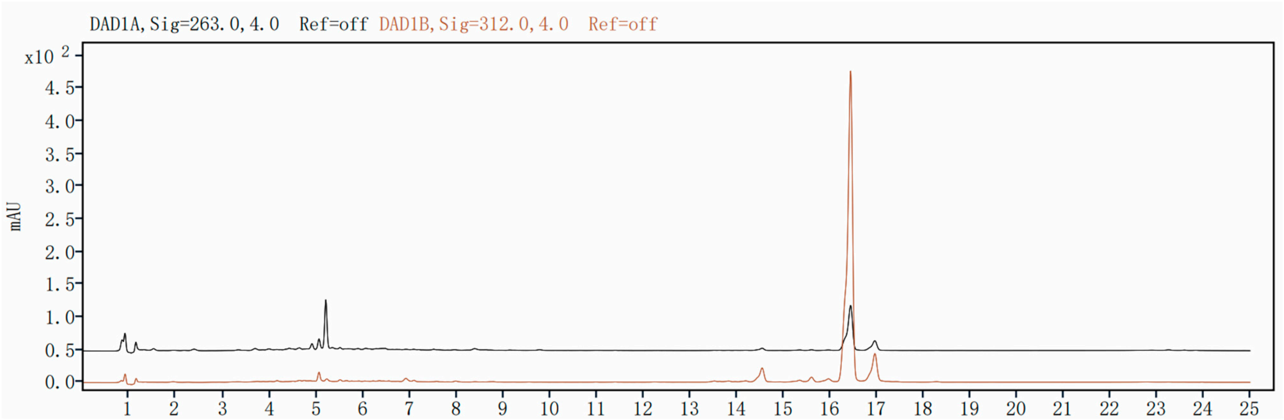

t/min

50% methanol

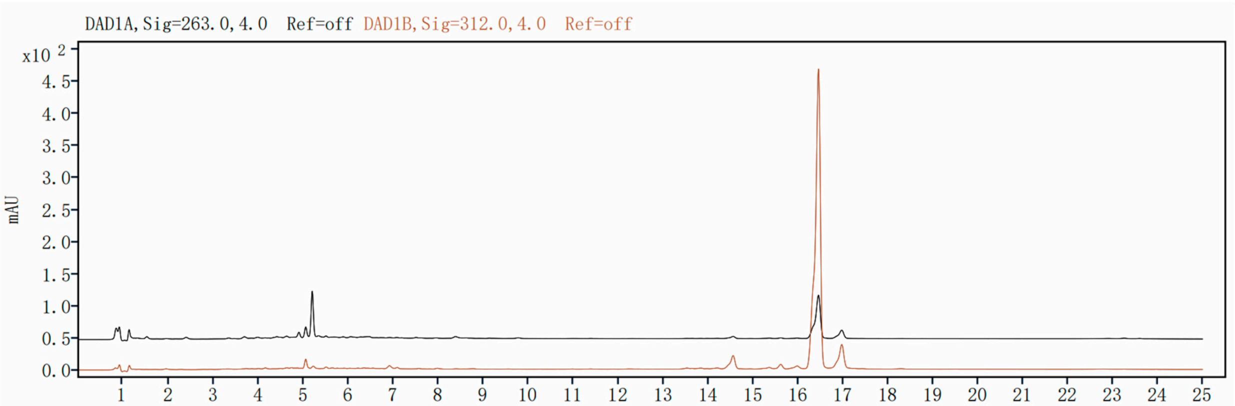

t/min

75% methanol

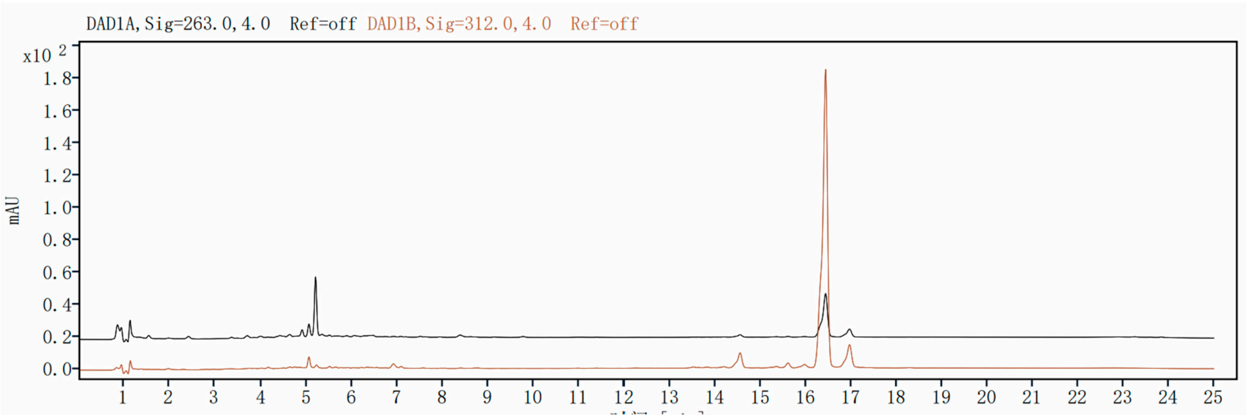

t/min

100%methanol

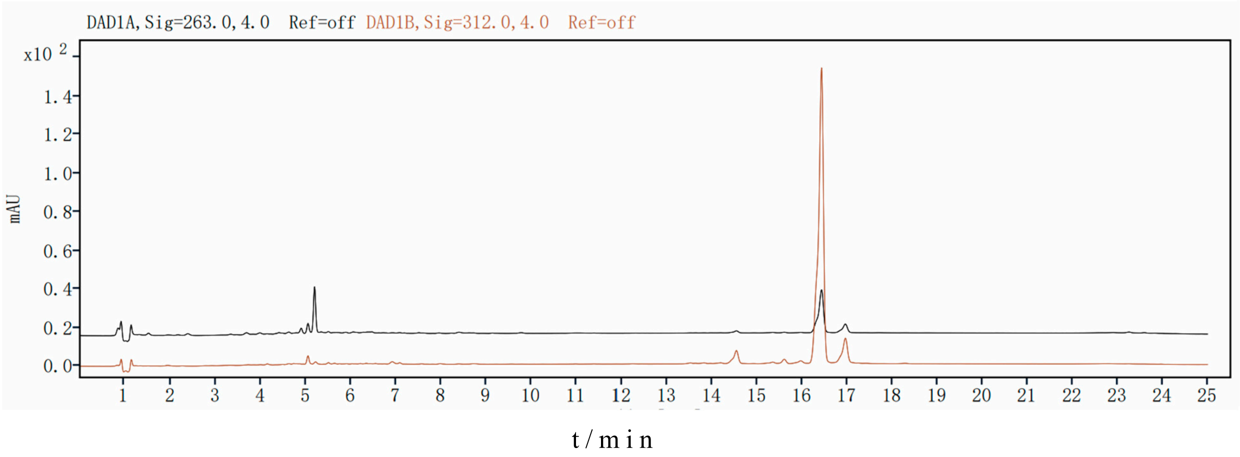

Extraction Time

30min

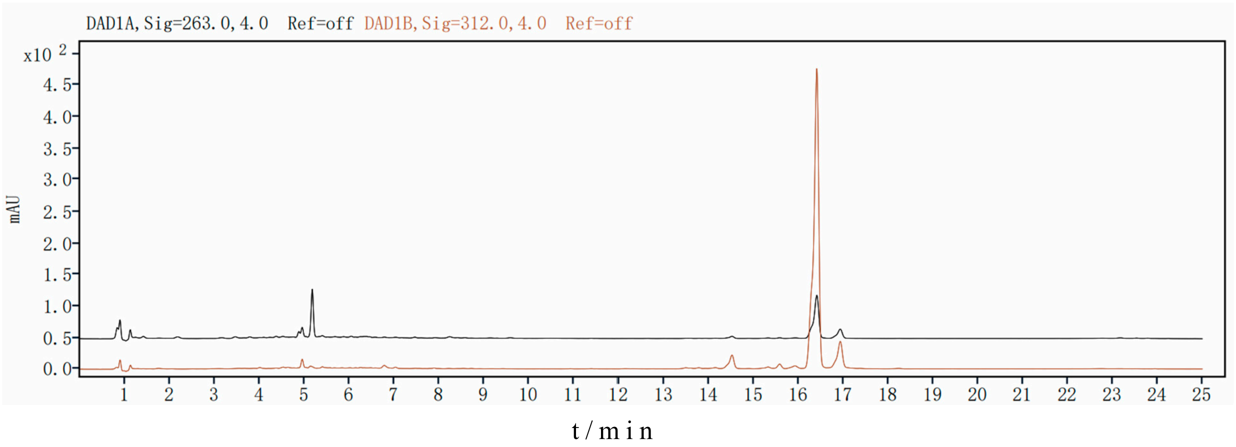

60min

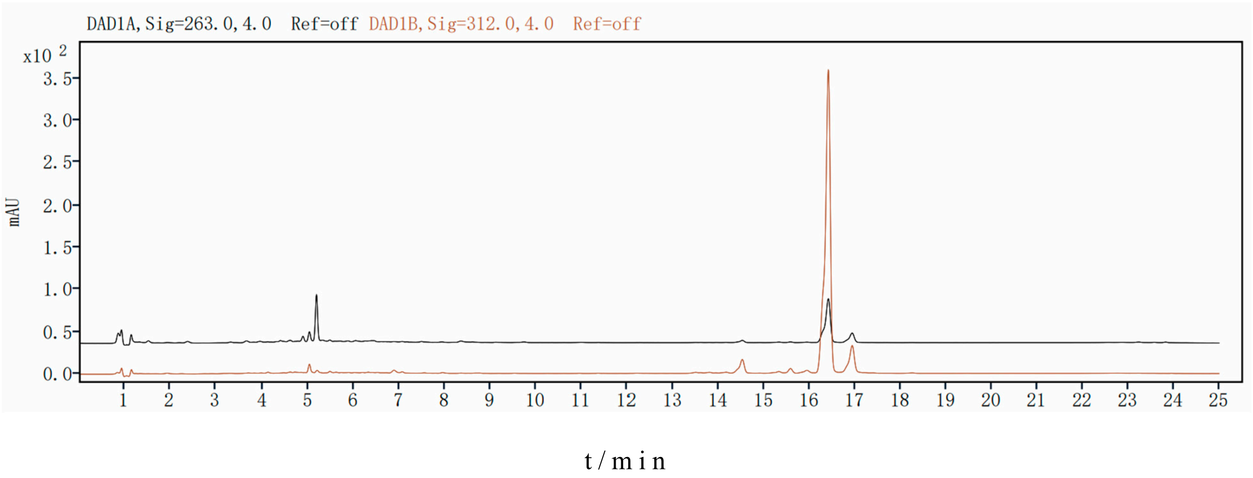

90min

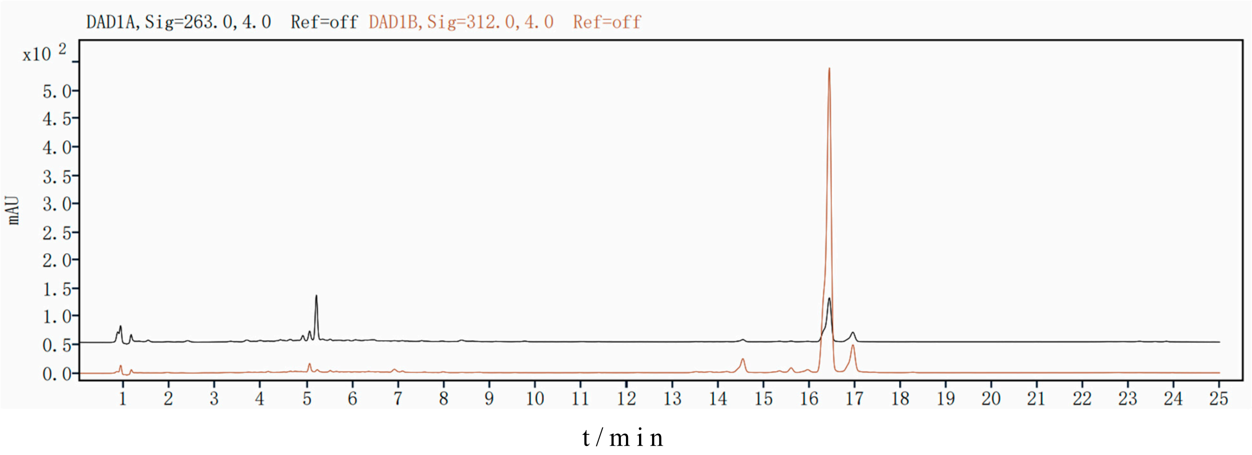

120min

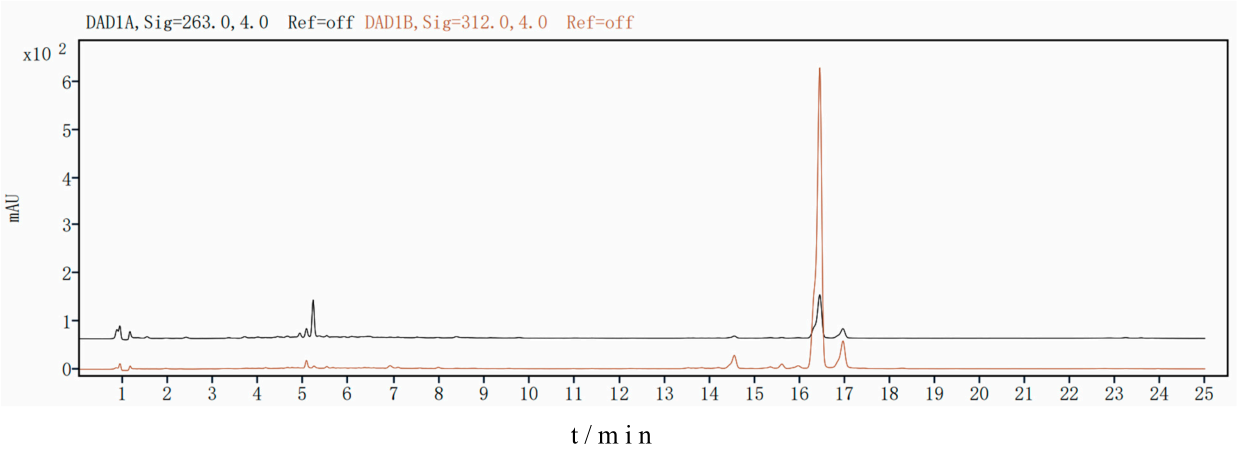

Material-liquid Ratios

(1:10 w/v)

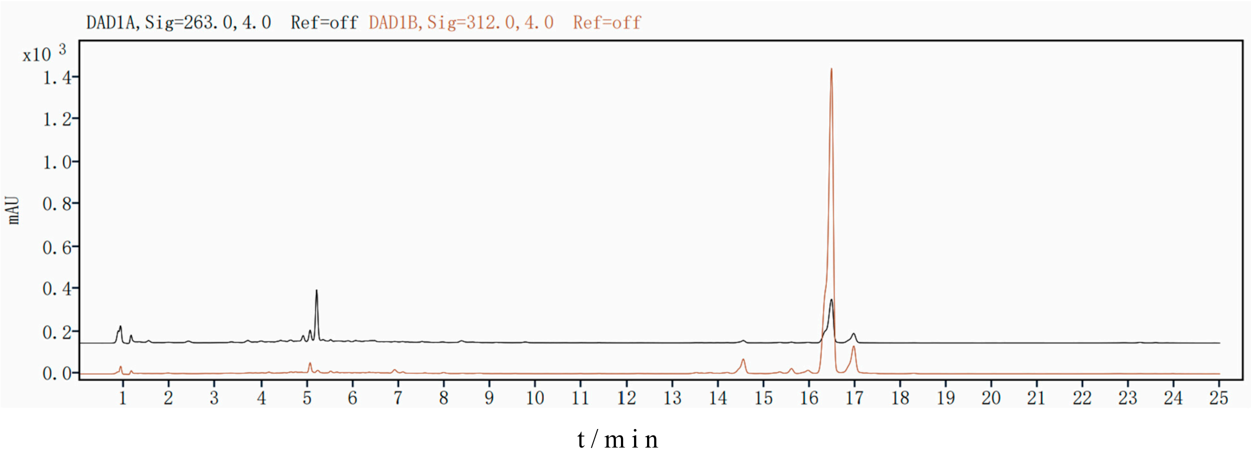

(1:25 w/v)

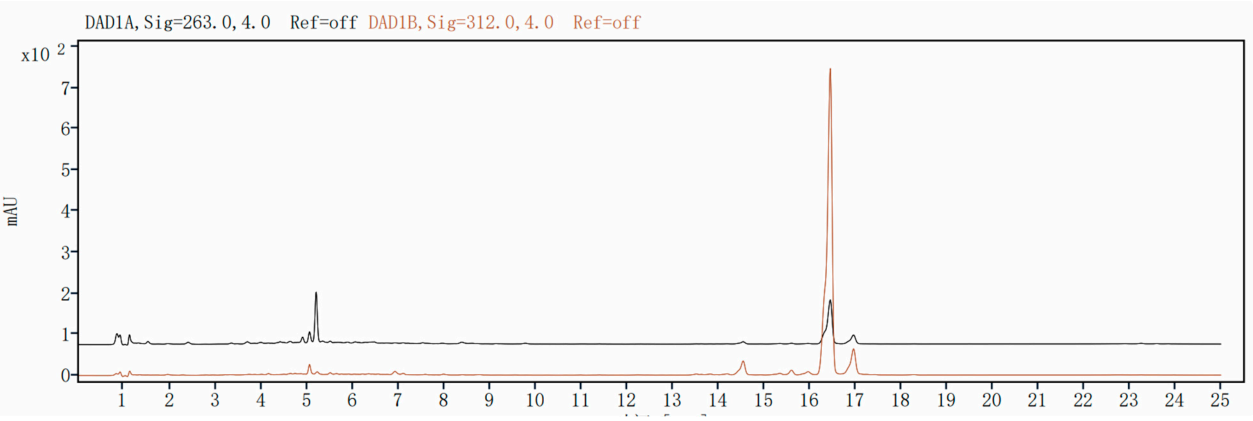

t/min

(1:50 w/v)

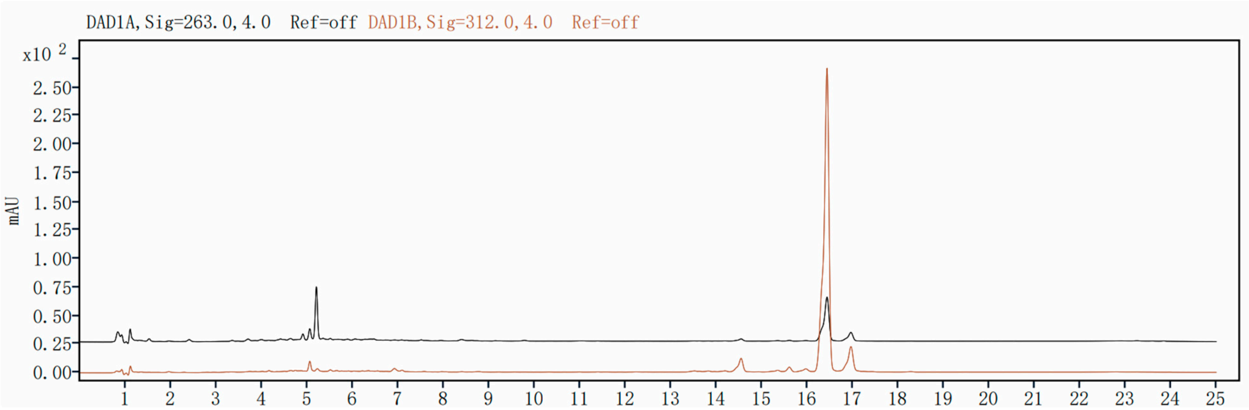

t/min

(1:100 w/v)

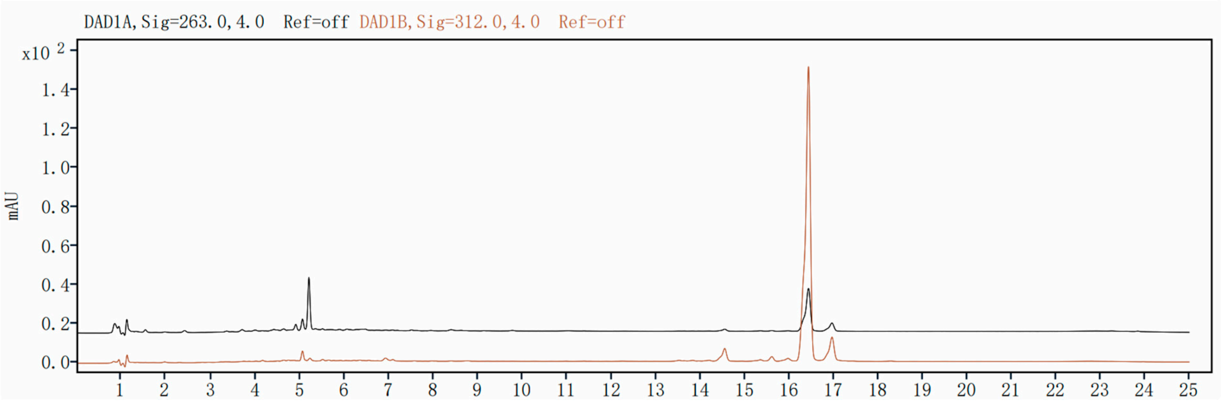

t/min
