# Supplementary material for: Comprehensive Evaluation of Quality and Differences in Silene viscidula Franch from Different Origins Based on UPLC-ZENO-Q-TOF-MS/MS Compounds Analysis and Antioxidant Capacity
Source: Molecules. 2024 Oct 11;29(20):4817. doi: 10.3390/molecules29204817 (PMC11509892; doi:10.3390/molecules29204817)

1. Arginine   74-97-3

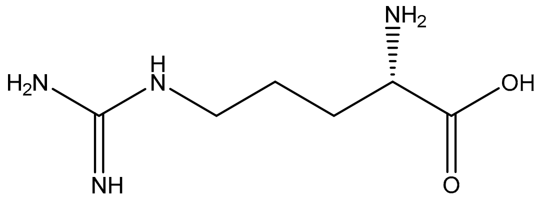

2. L-5-Hydroxytryptophana   268738-23-4

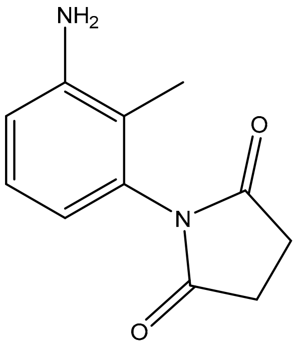

3.   siliendine A   1417825-84-3

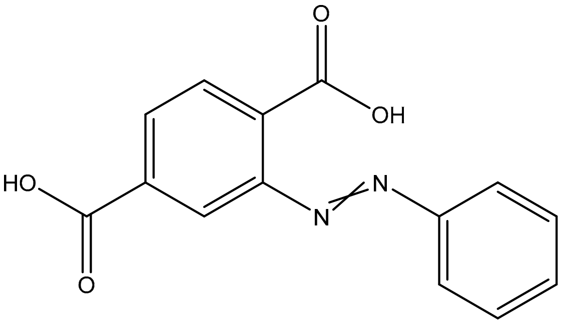

4. Proline   344-25-2

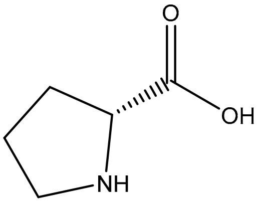

5. 3β-acetoxy-(25R)-5α-spirostan-12-onea 1772614-25-1

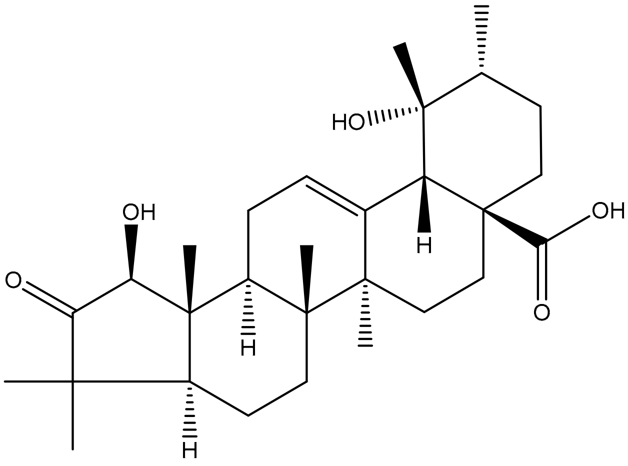

V

6.Tyrosine 60-18-4

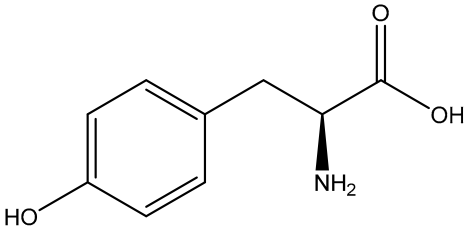

7. Coumarin CB6775470

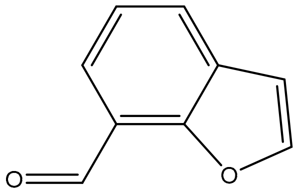

8. Isoleucine 7004-09-3

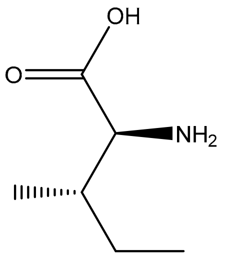

9. 1,2,3,4-Tetrahydro-1H-pyrido[3,4-b] indole-3-carboxylic Acida 481710-98-9

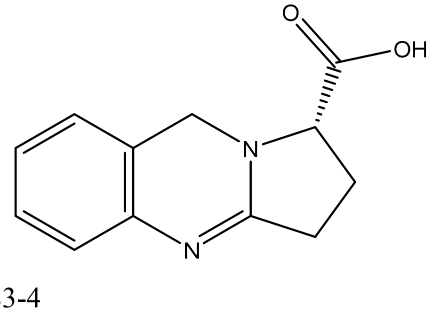

10. Pantothenic acid 79-83-4

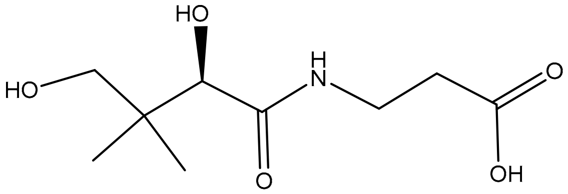

11. N-(1-Deoxy-1-fructosyl)Tryptophan 2230887-20-2

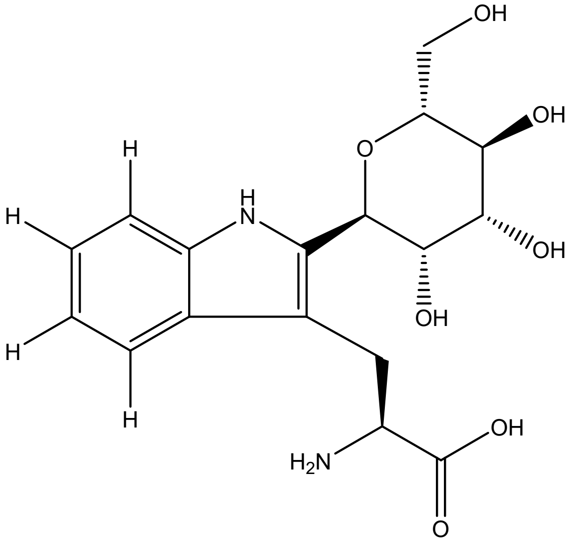

12. trans-3-Indoleacrylic acid 29953-71-7

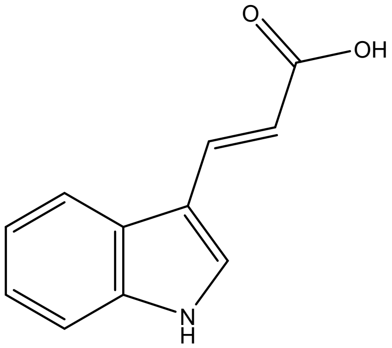

13. Licoagroside B 325144-72-7

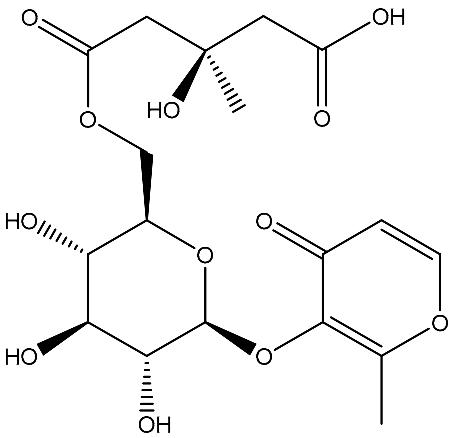

14. Macrostemonoside I 145854-04-2

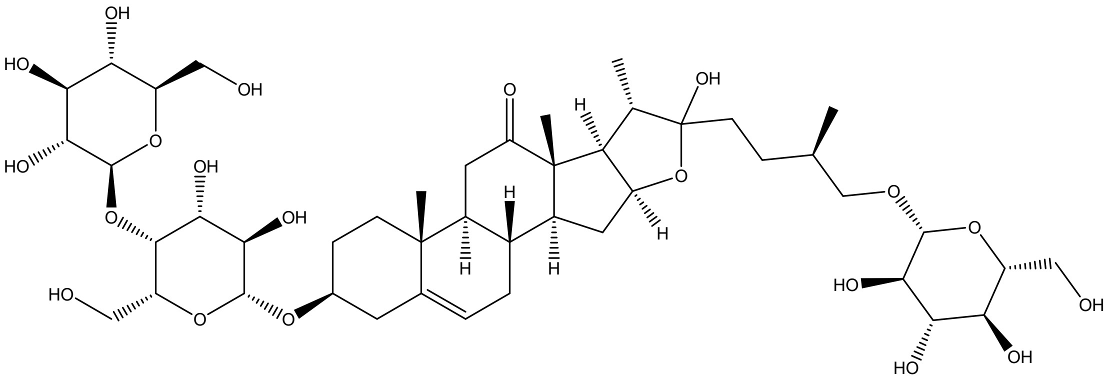

15. Vincetoxicoside A 1416-01-9

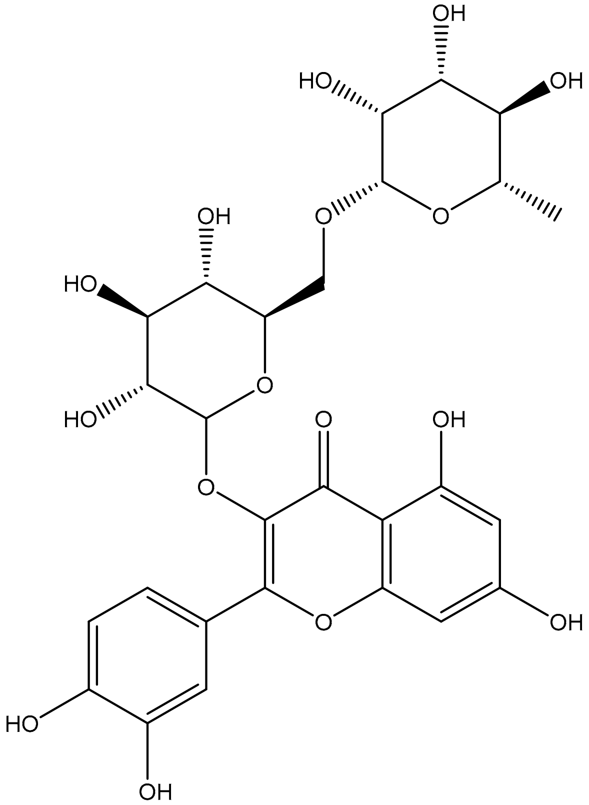

16. Apiin 1426544-34-3

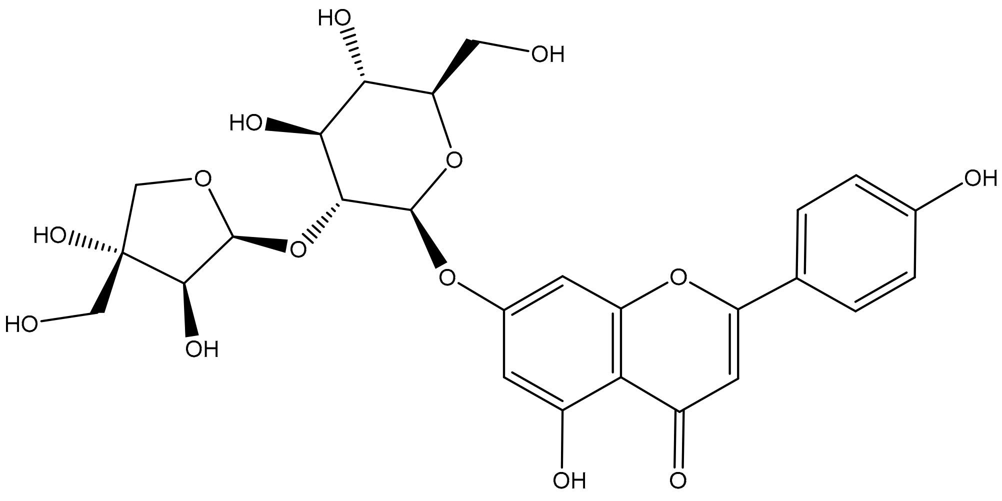

17. Pregn-5-en-3β,20(S)-diol-3-O-bis-β-D-glucopyranosyl-(l-2,1-6)-β-D-glucopyranoside
2568203-95-0

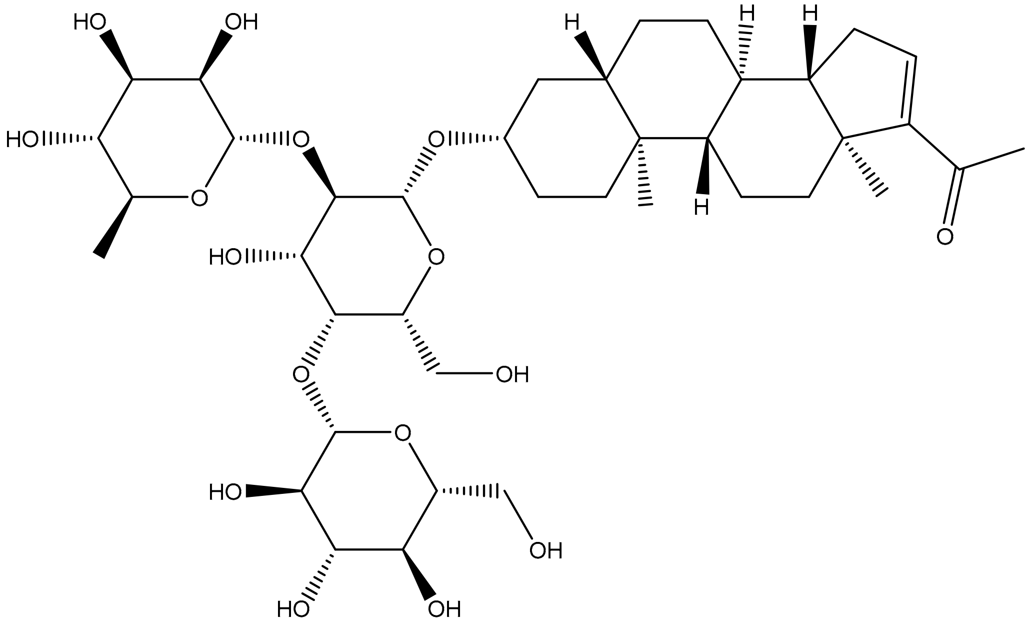

18. tupistroside K 109771-10-0

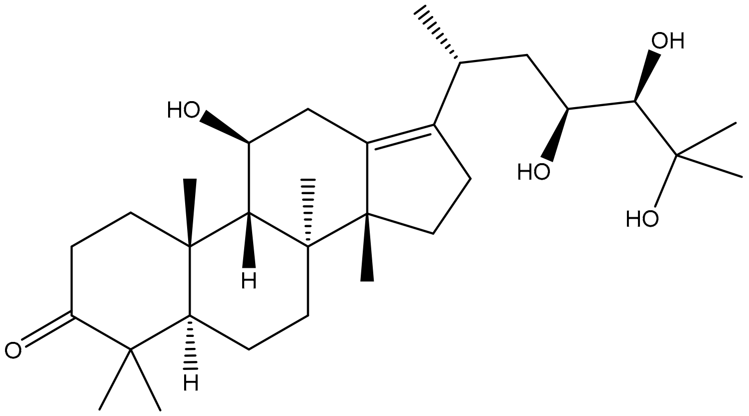

19. Ecdysterone 2,3-Monoacetonide 22-O-Benzoate 39012-22-1

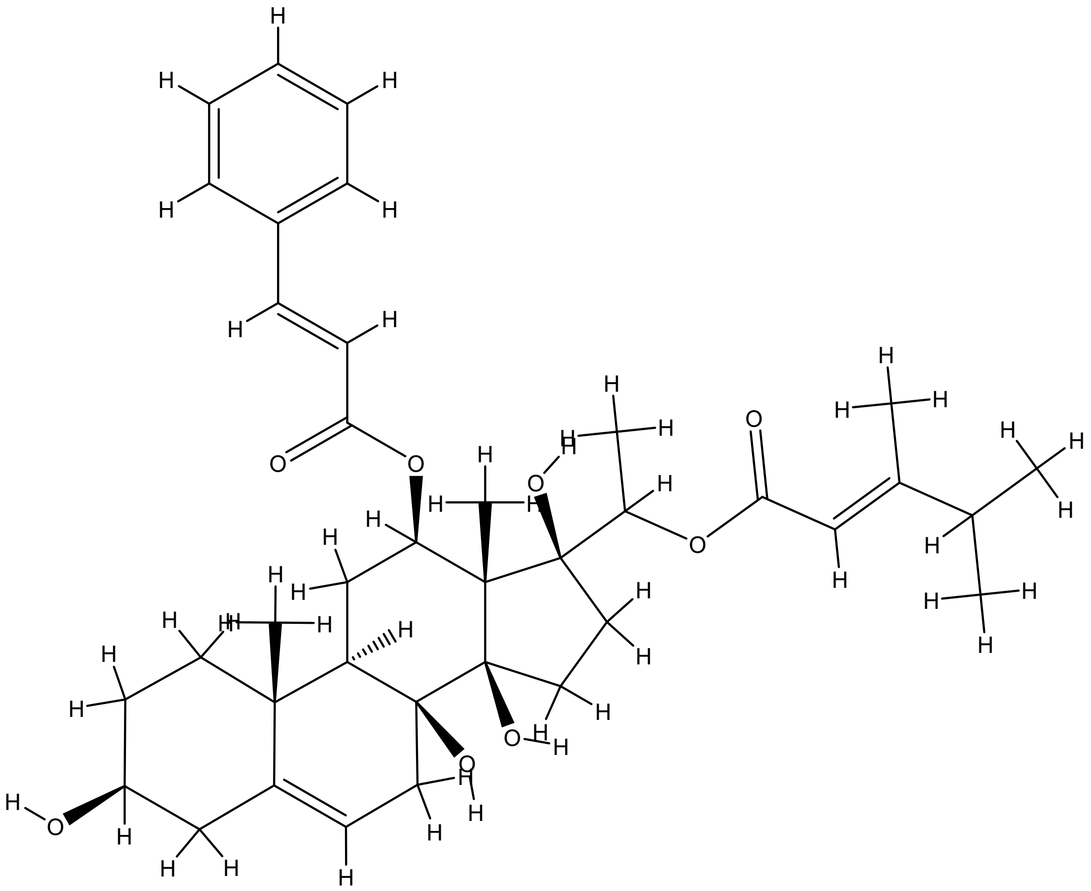

20. siliendine C PubChem CID 135976670

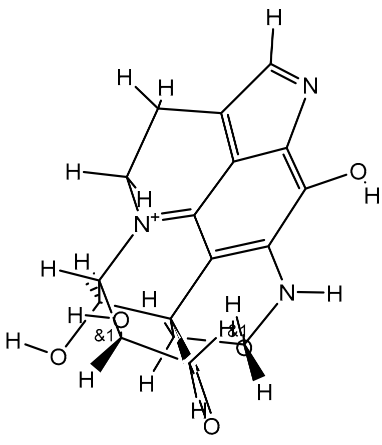

21. sileneoside G 1384968-72-2

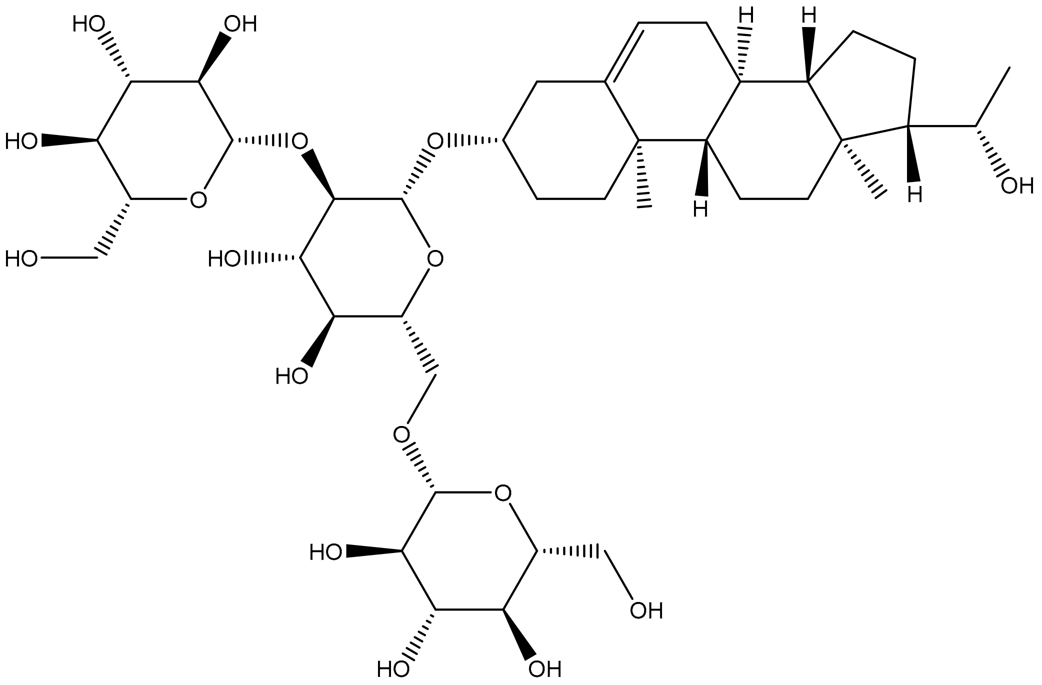

22. Oroxin B 23666-13-9

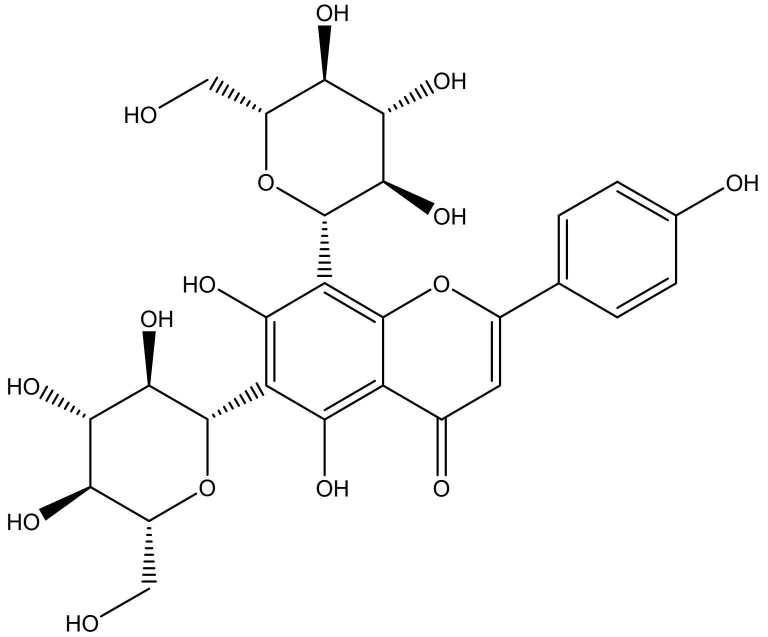

23. Ophiopogonin R 937181-26-5

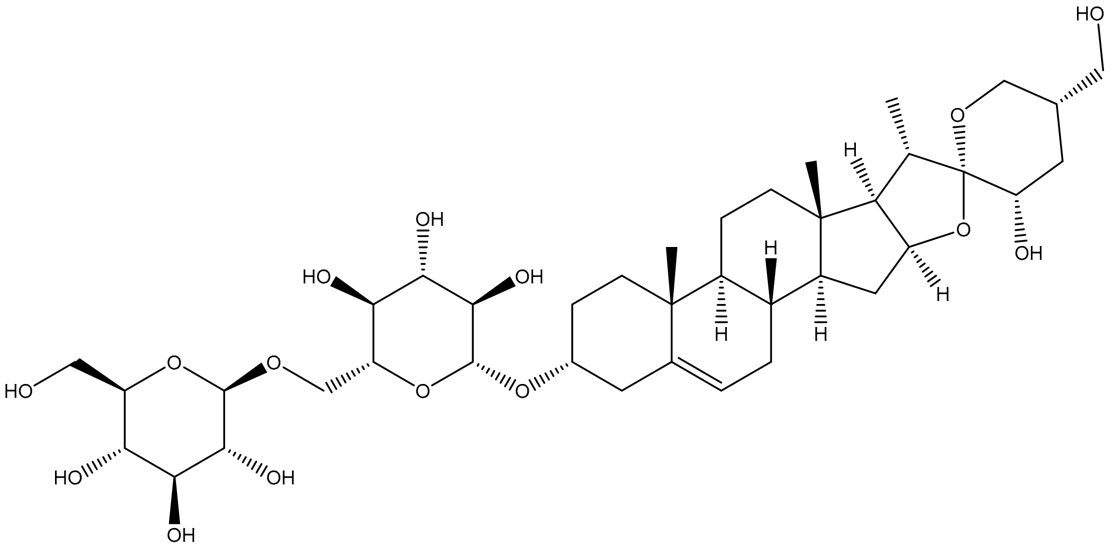

24. tupistroside L   172046-43-4

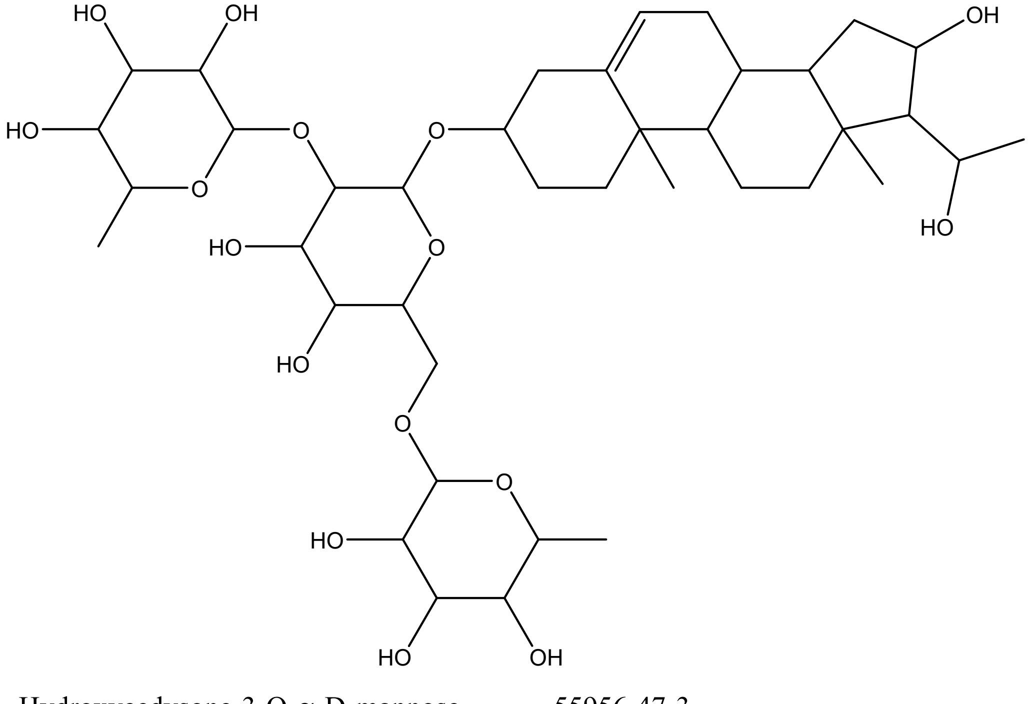

25.   Hydroxyecdysone-3-O-α-D-mannose   55956-47-3

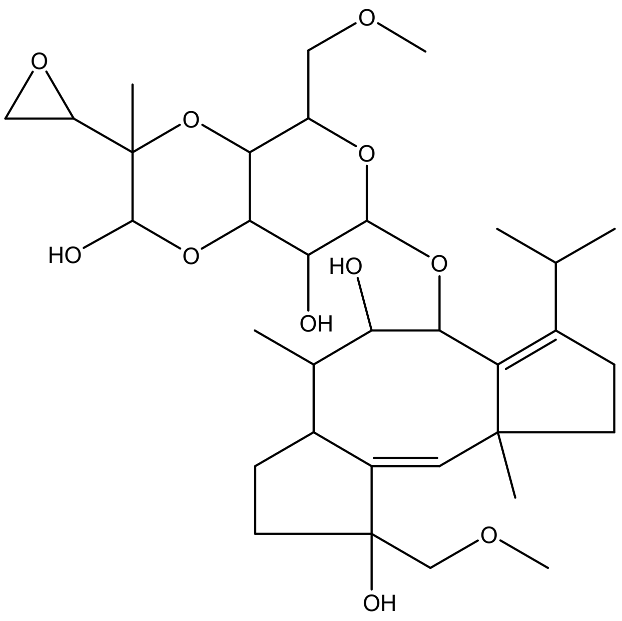

26. tupistroside J   521970-06-9

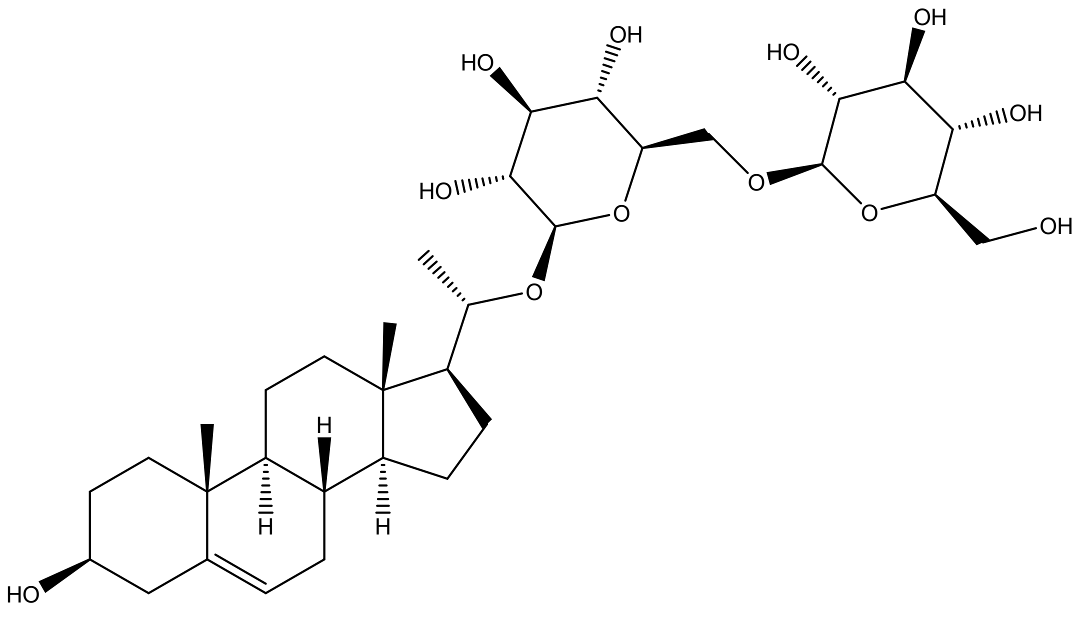

27. Caucasicoside A   81058-28-8

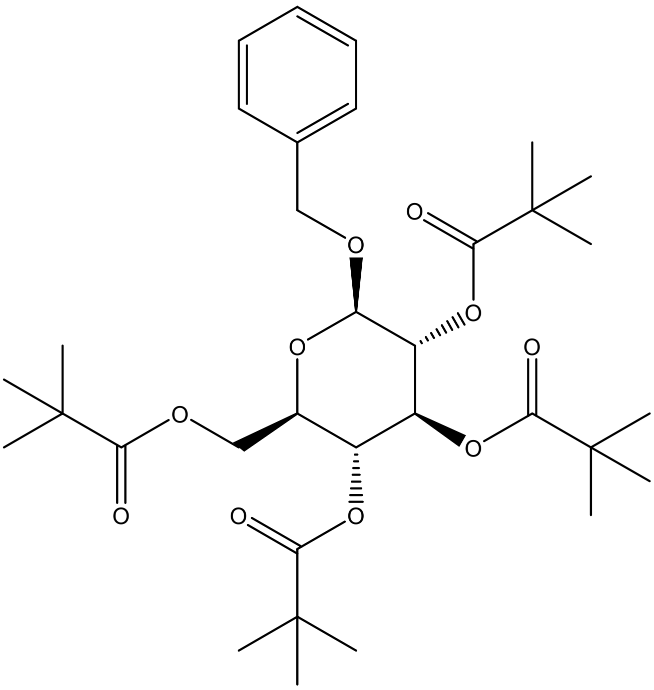

28. dehydro-8-gingerdione 19466-41-2

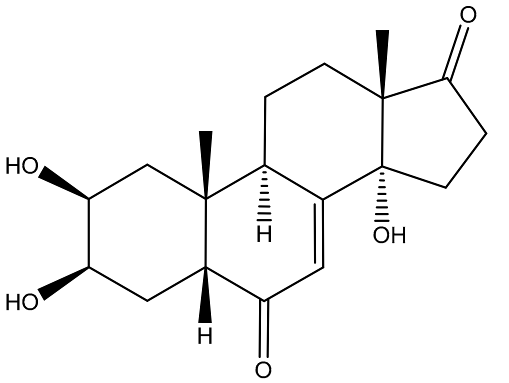

29. Polyporoid B 1042362-45-7

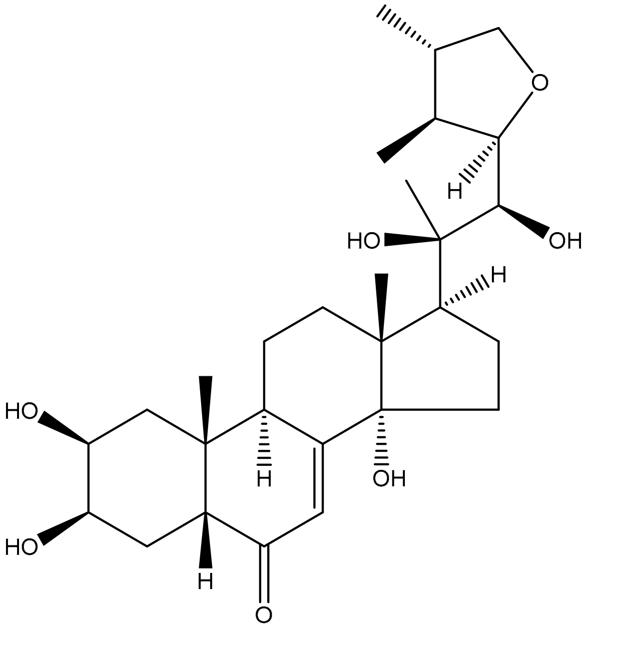

30. 26-Hydroxypolipodine B CB31389914

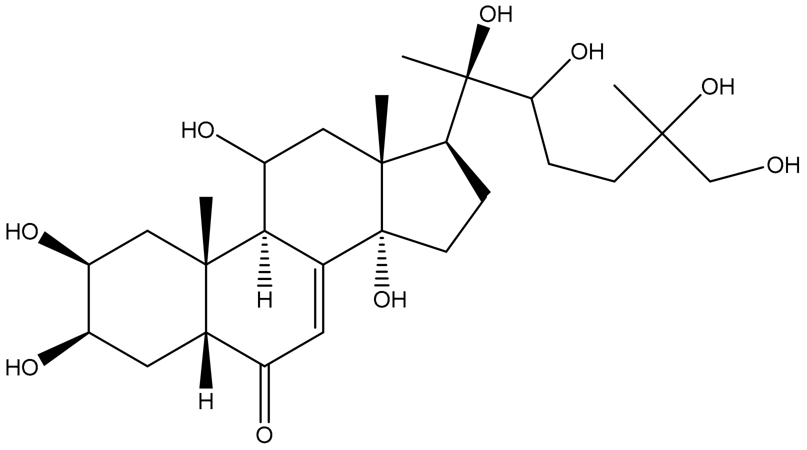

31. 26-Hydroxyecdysone 5289-74-7

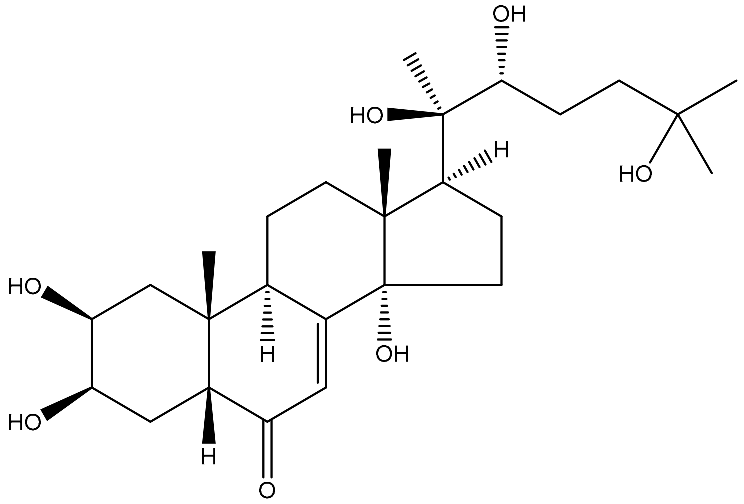

32. 3-dehydroecdysone 83921-17-9

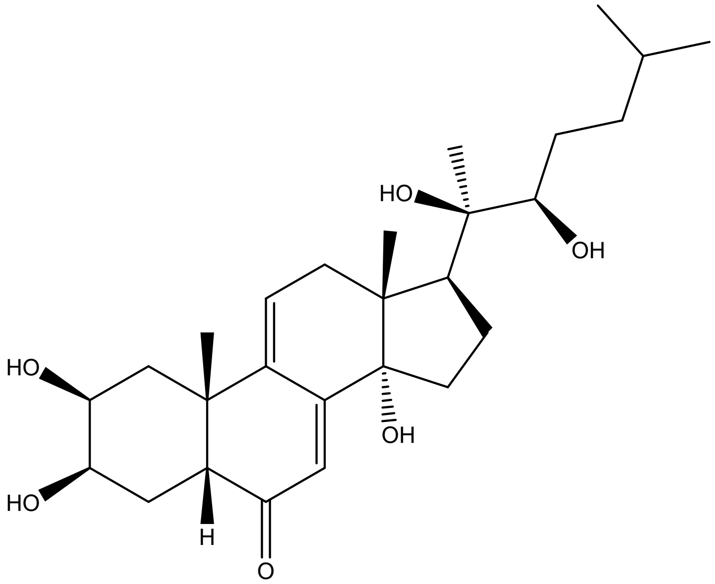

33. armeroside B 142674-88-2

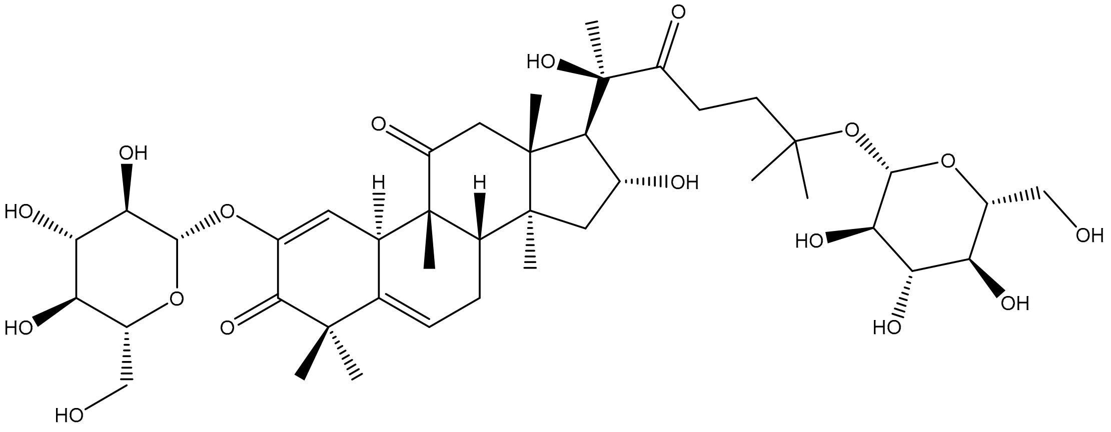

34. Cosmosiin (Apigenin-7-Oglucoside) 578-74-5

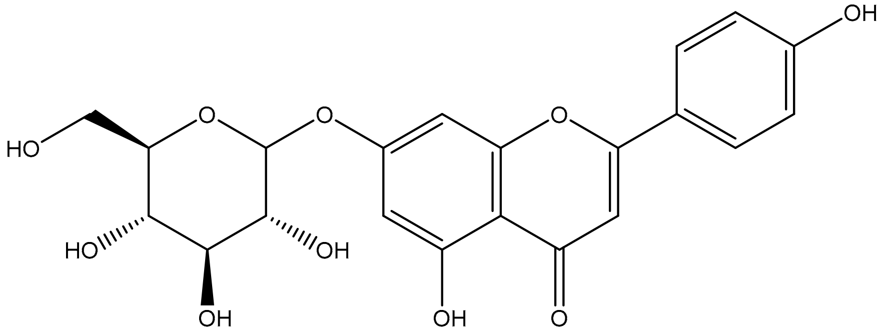

35. Abutasterone 19458-46-9

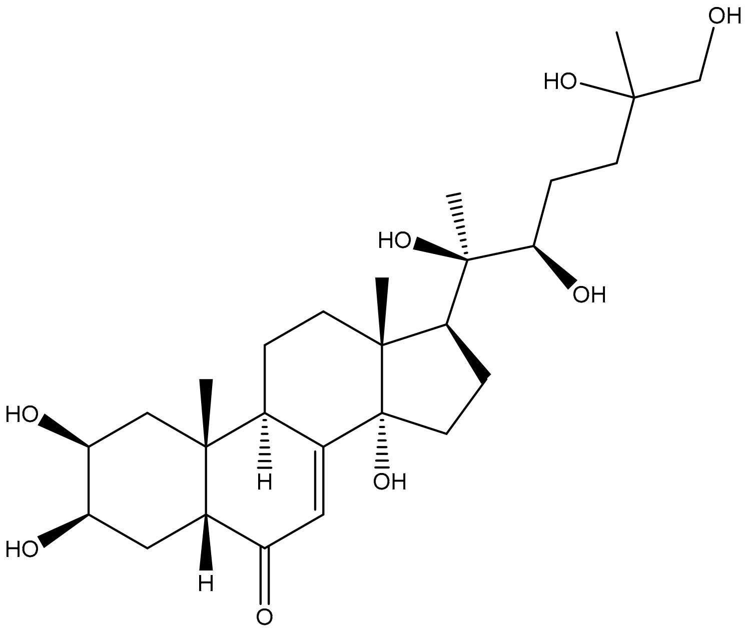

36. Ecdysteroid 586960-44-3

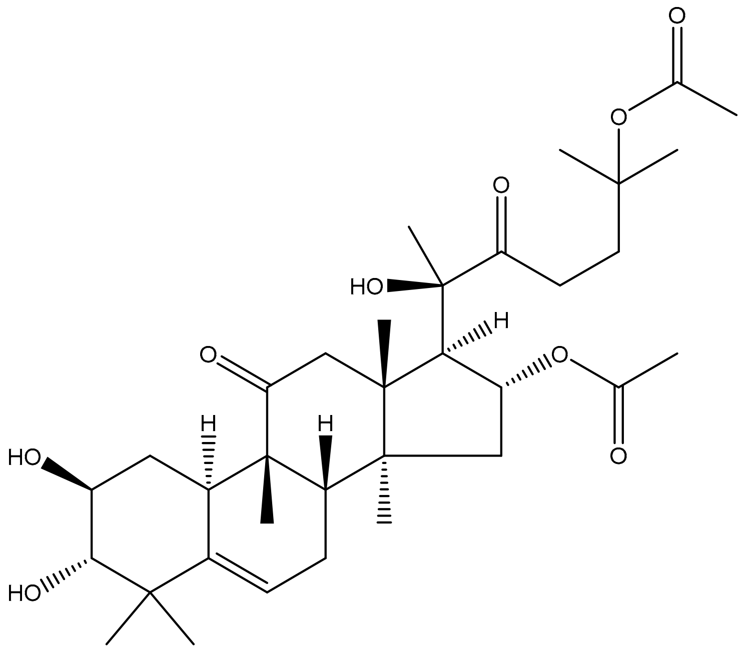

37. 2-Dehydroxyecdysterone-3-O-benzoate 54614-58-3

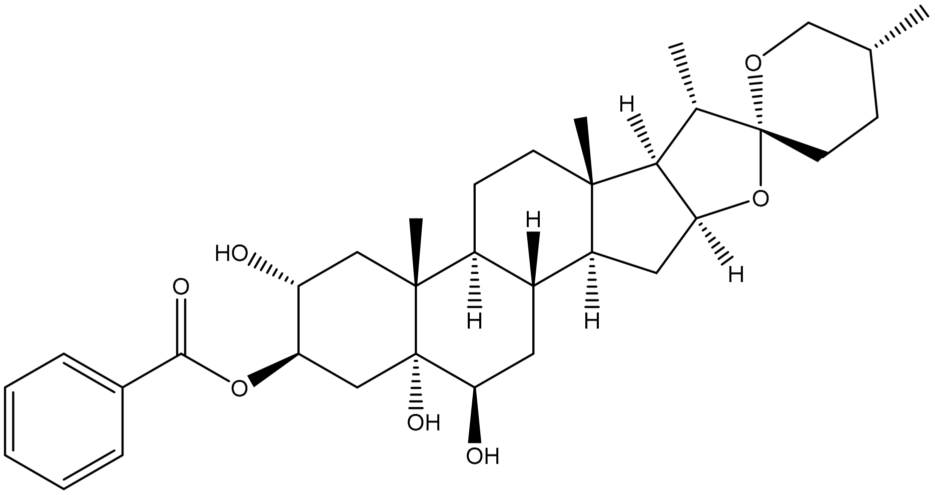

38. Chrysoeriol-7-O-glucoside 19993-32-9

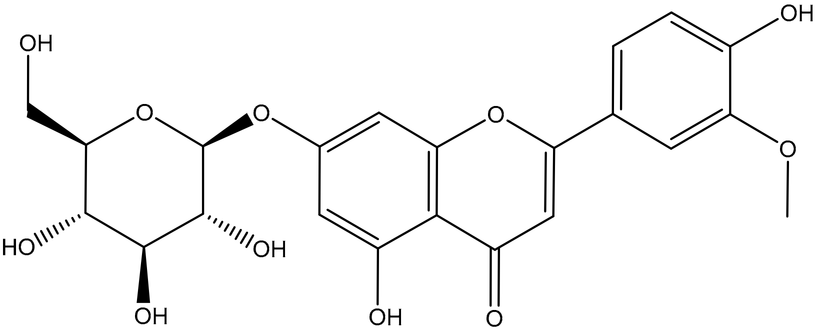

39. Hydroxyecdysone-3-O-α-D-mannose-O 1373440-76-6

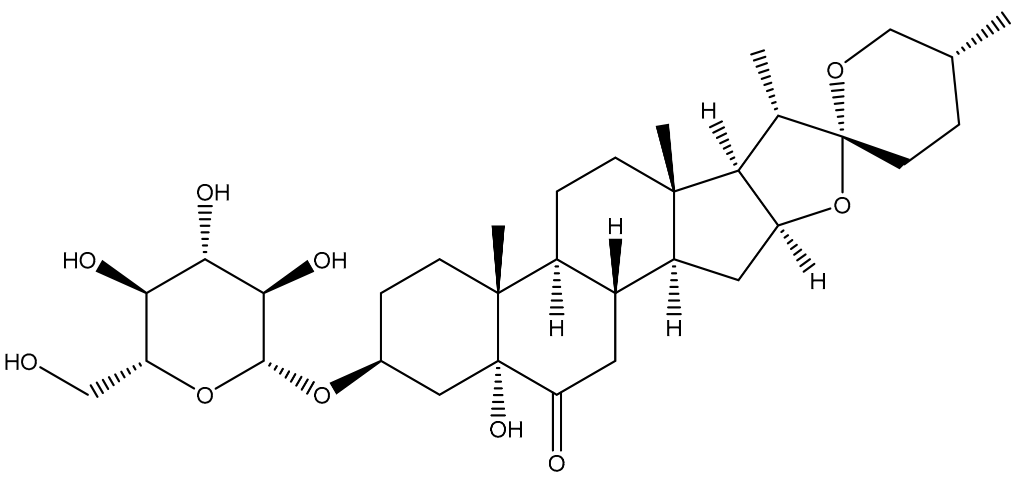

40. Hydroxyecdysone-3-O-α-D-mannose+CH_2CH_3 PubChem CID 11082916

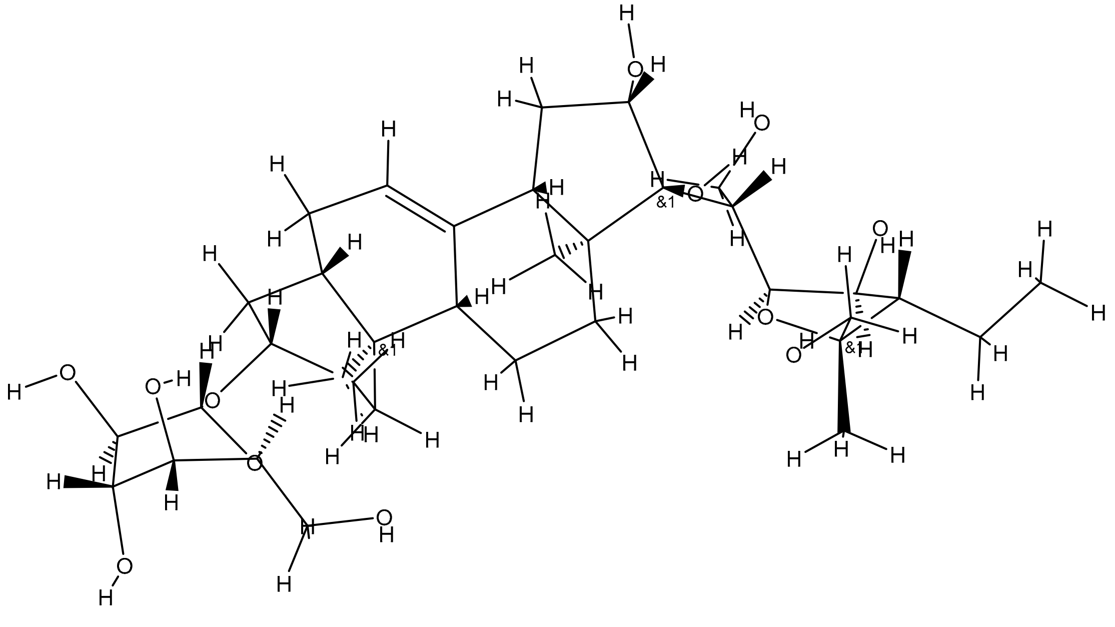

41. Makisterone A 20137-14-8

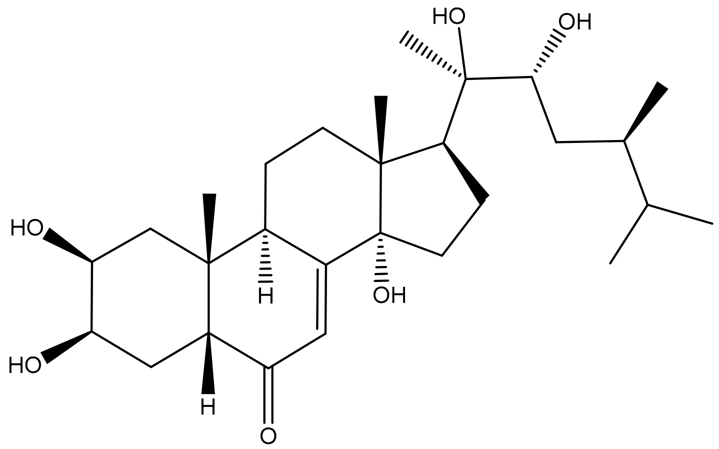

42. Ponasteroside A 20117-33-3

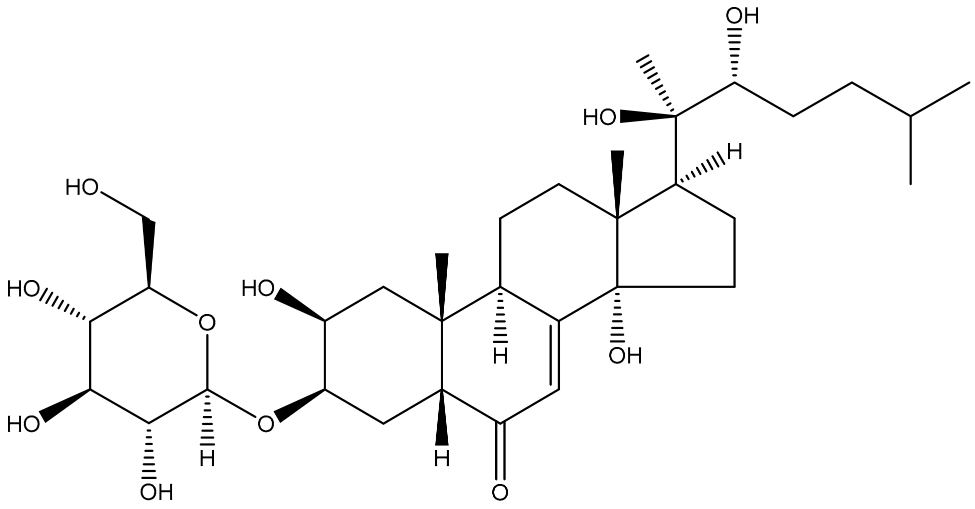

43. Desonide 638-94-8

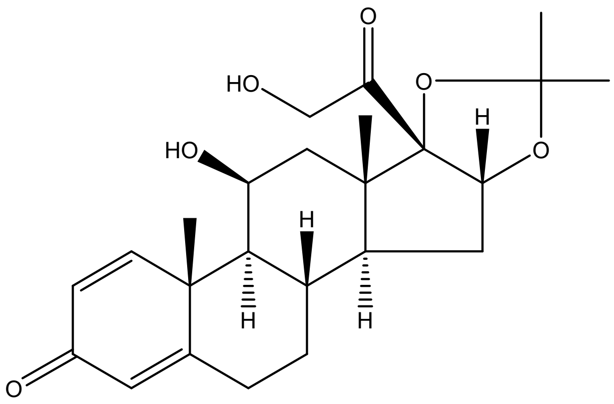

44. 2-Deoxyecdysone 22β-D-glycosidea 651306-80-8

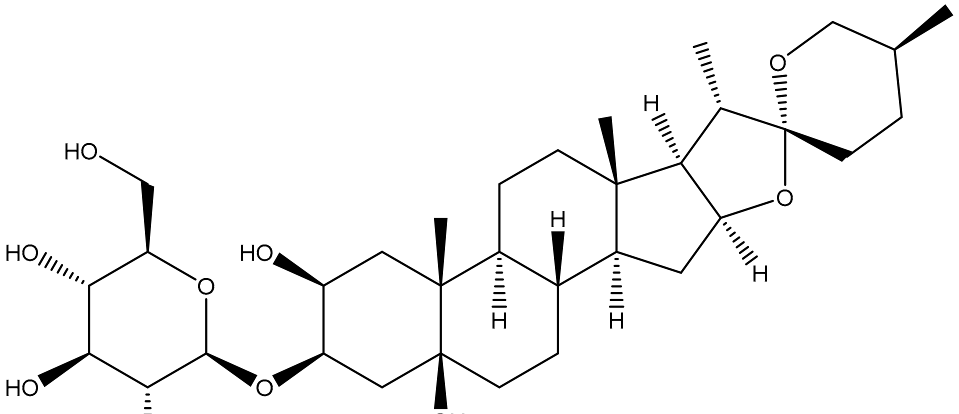

45. Diosgenin 512-04-9

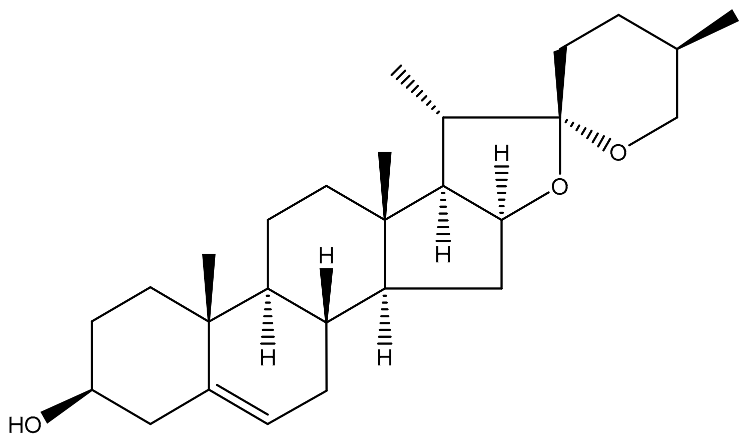

46. Viticosterone E 22033-96-1

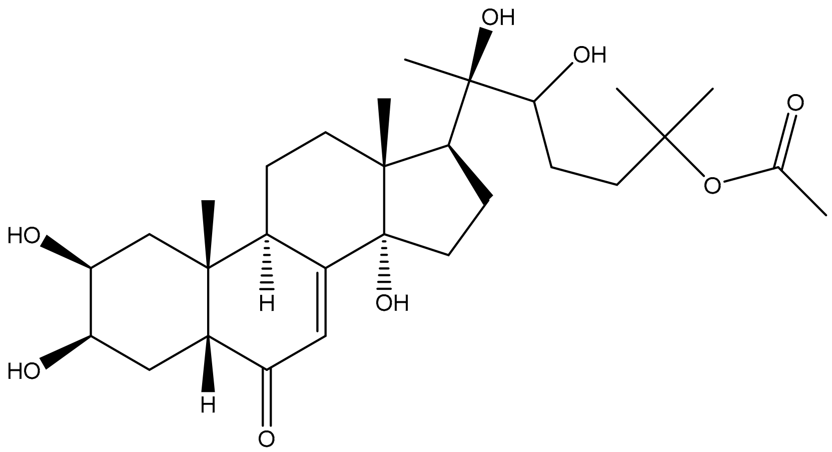

47. Lucidenic acid C 95311-96-9

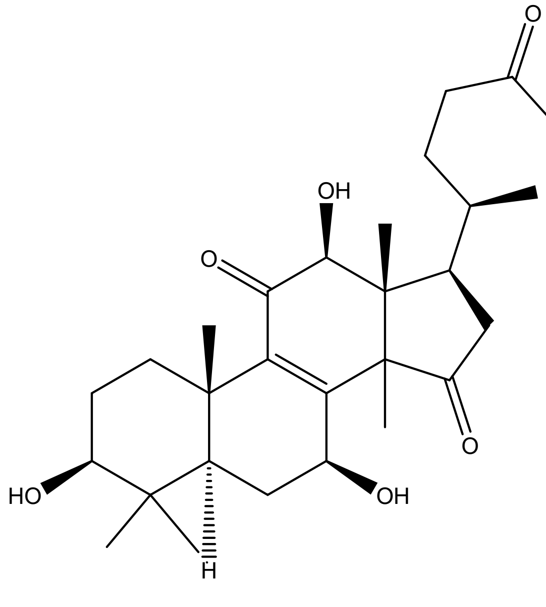

48. Ponasterone A 17146-23-5

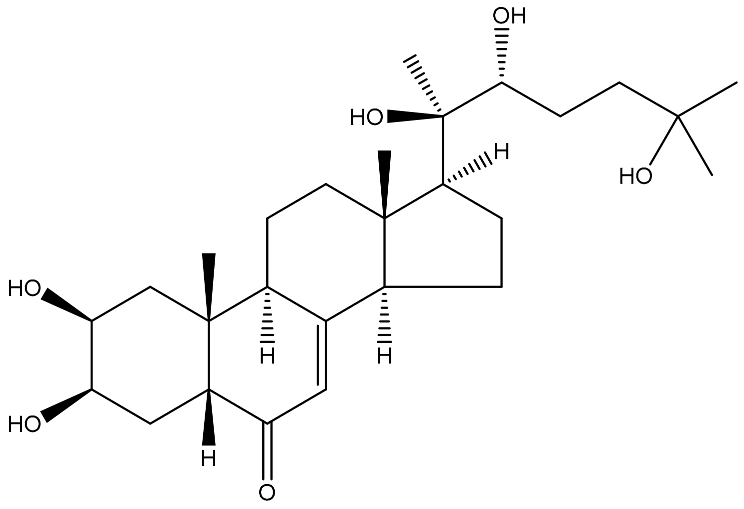

49. Makisterone C 245323-24-4

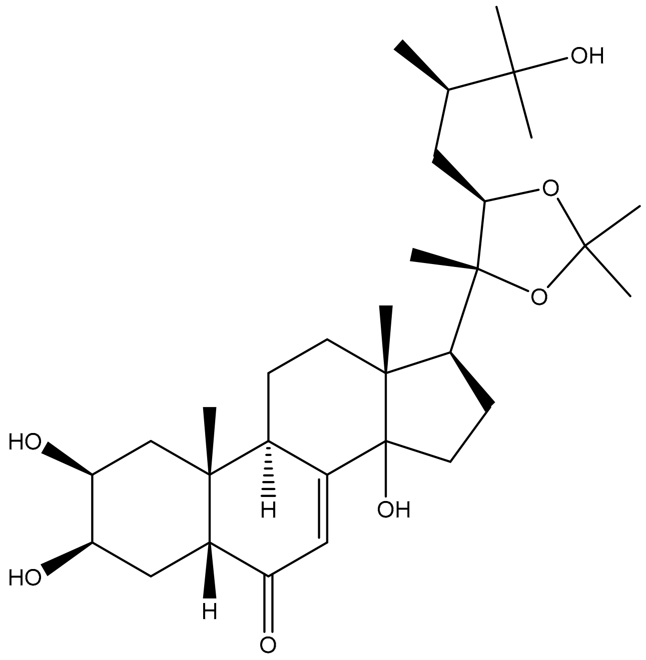

50. silenegallisaponin B 142846-87-5

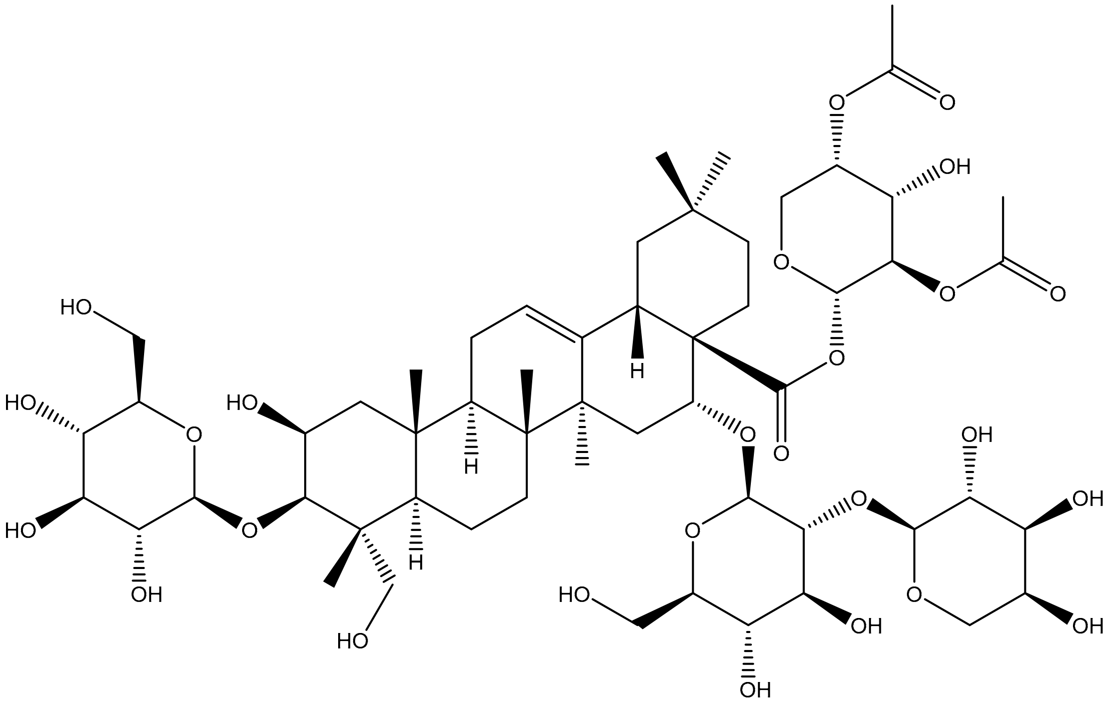

51.   Sileneoside B -diacetonide   1072072-37-7

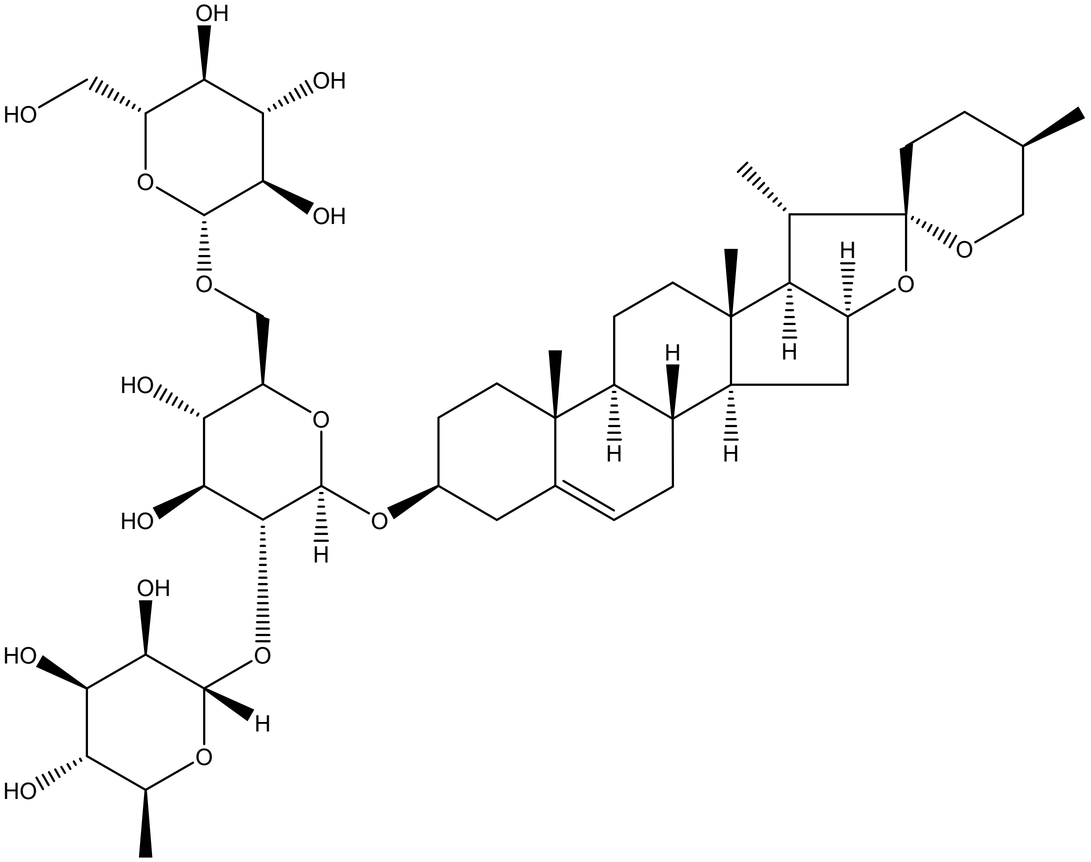

52. a-Ecdysone 2,3,25-Triacetate   PubChem CID   171326229

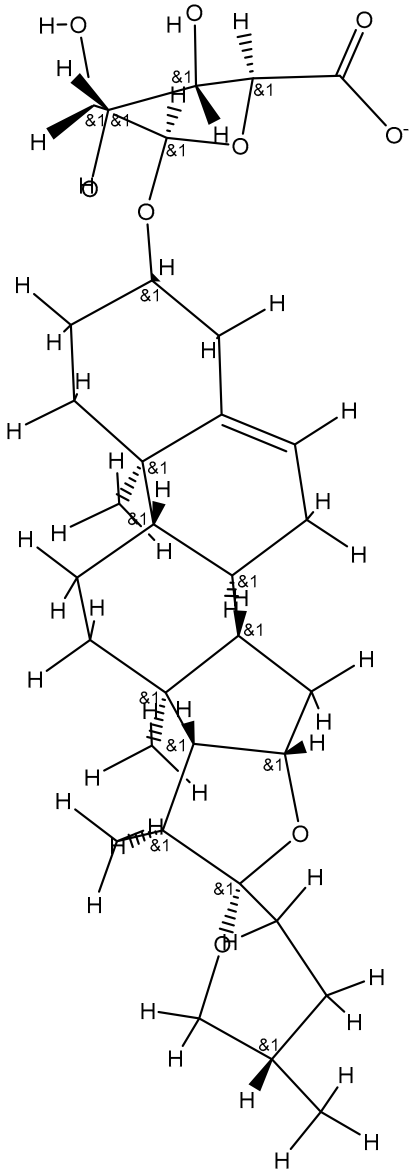

53. QUDA-GlcA-Ara/Xyl 178535-51-8

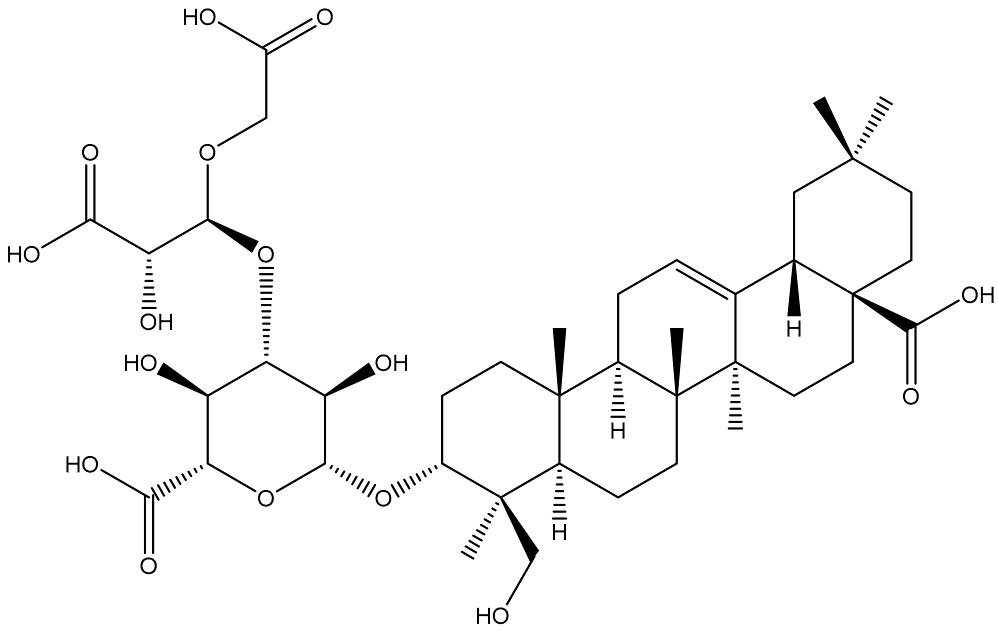

54. Armeroside D 141890-69-9

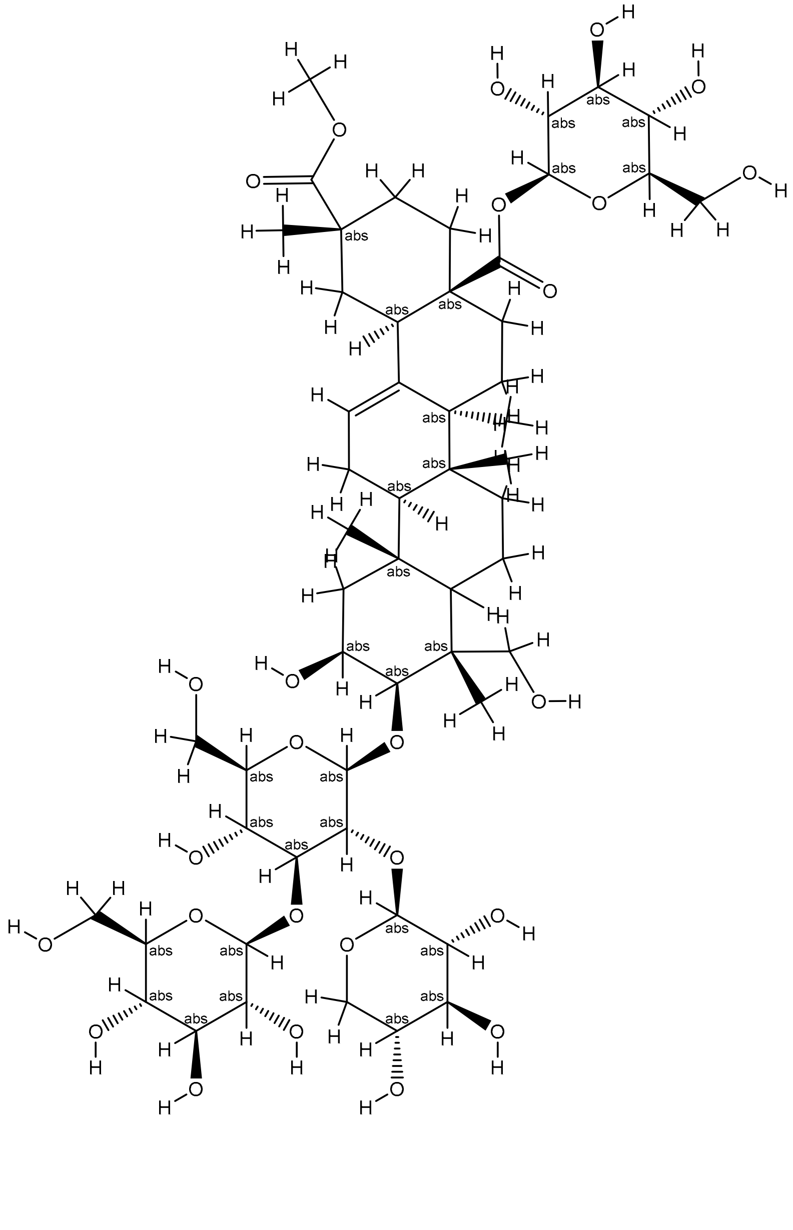

55. Licorice saponin A_3 215728-78-2

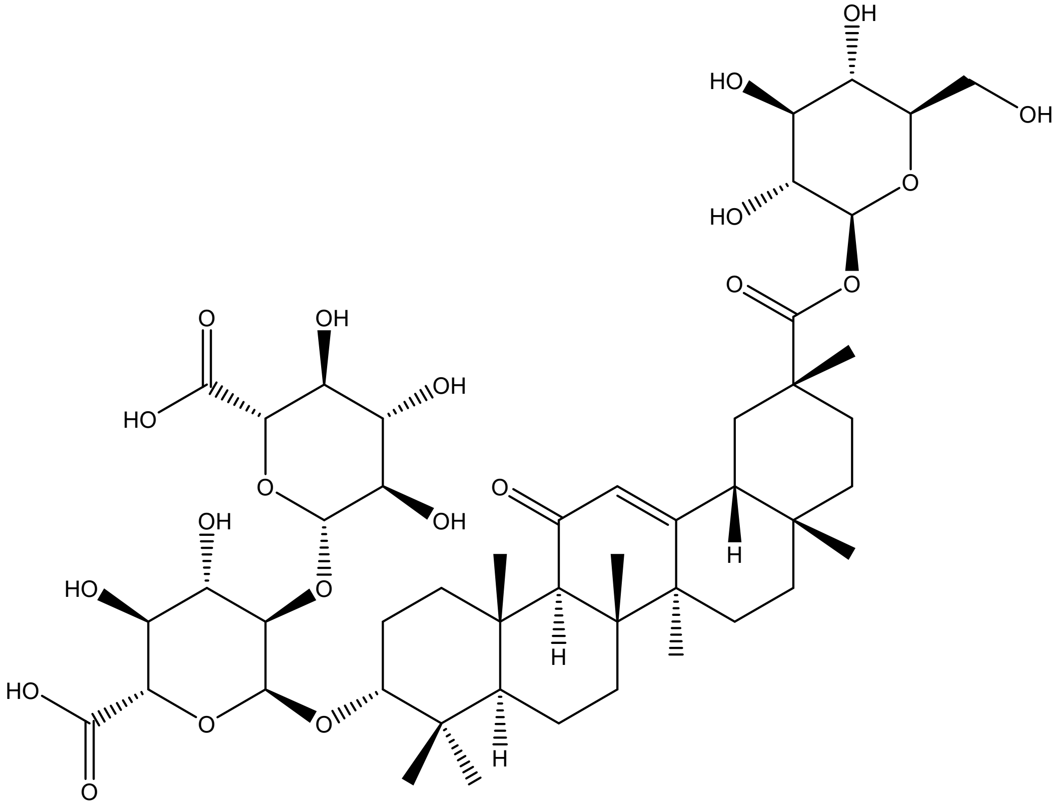

56. ecdysterone 20,22-monoacetonide 19885-10-0

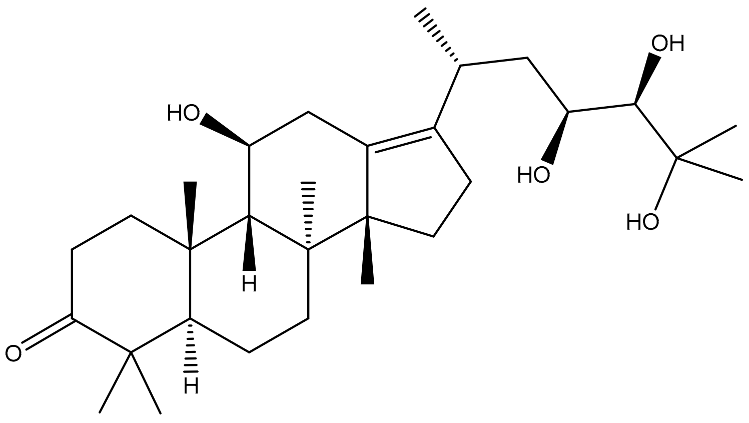

57. Dracaenoside C 4336-95-2

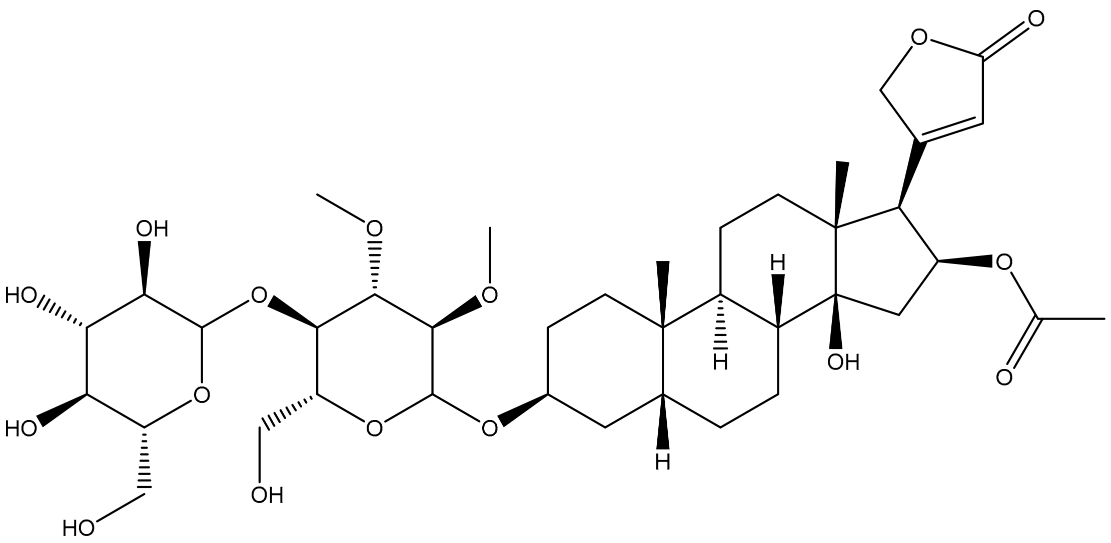

58. 3β-16β-dihydroxy-olean-12-ene-23,28-dioic acid   114567-48-5

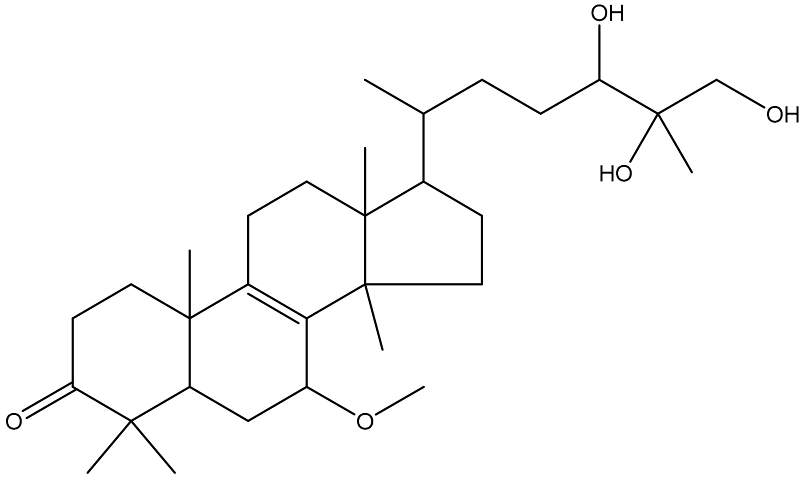

59. Ecdysterone 22,25-Di-O-benzoate   2802427-04-7

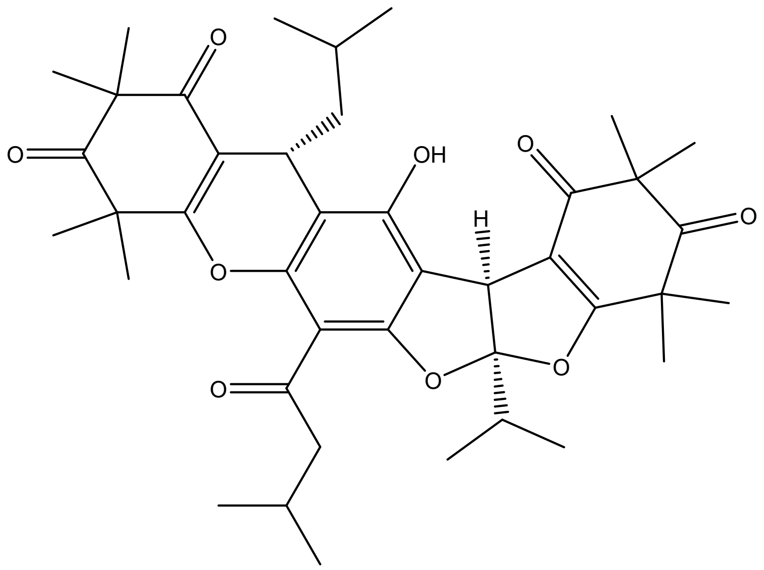

60. sinocrassuloside I   91652-23-2

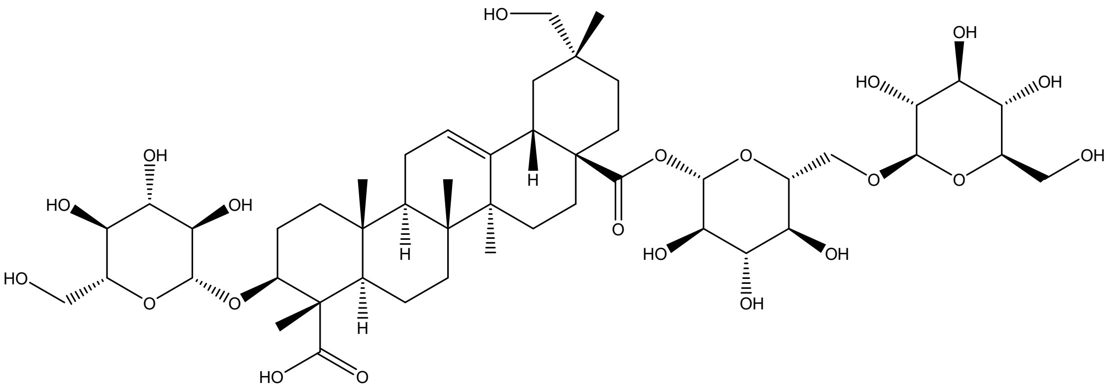

61. Dianchinenoside D 65497-07-6

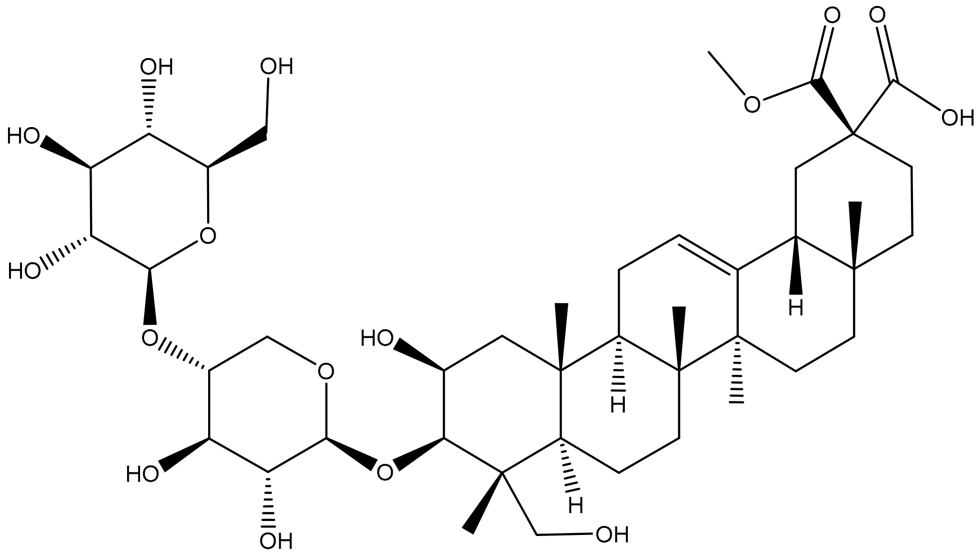

62. α-Humulene 1174496-34-4

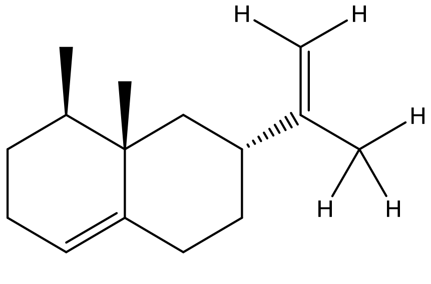

63. Tomentesterone B 181939-54-8

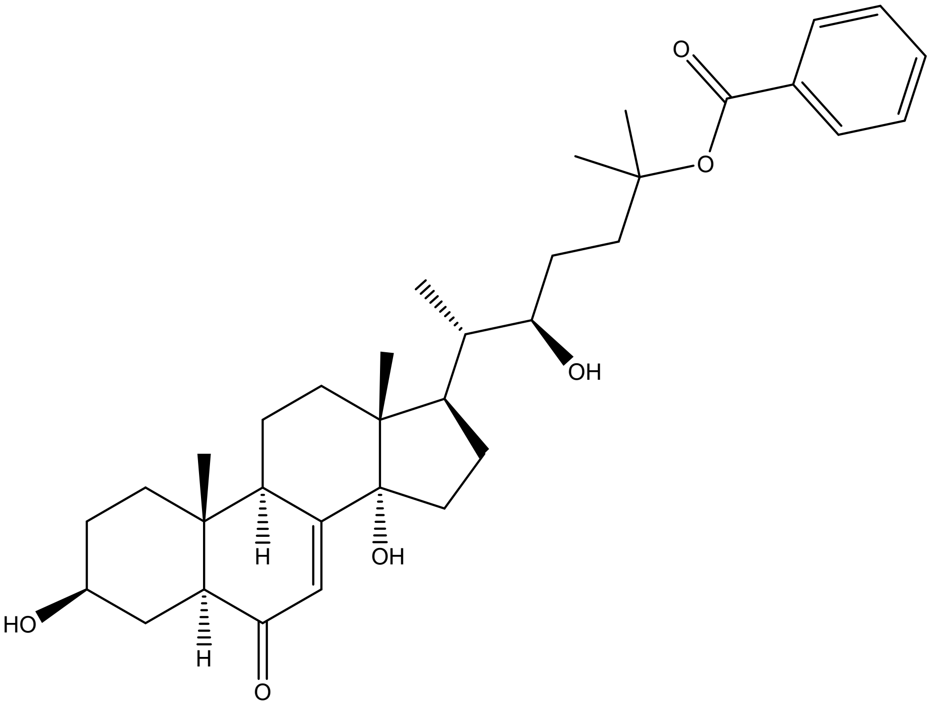

64. 4-Dehydroecdysterone 6199-84-4

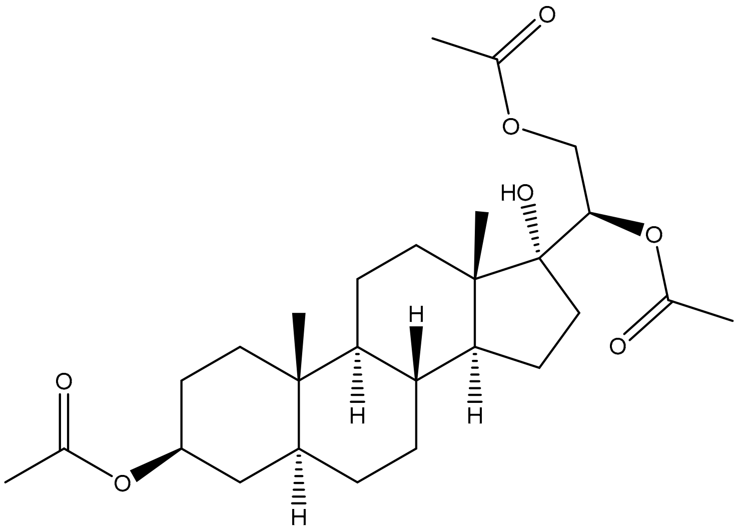

65. Kingianoside A 145854-03-1

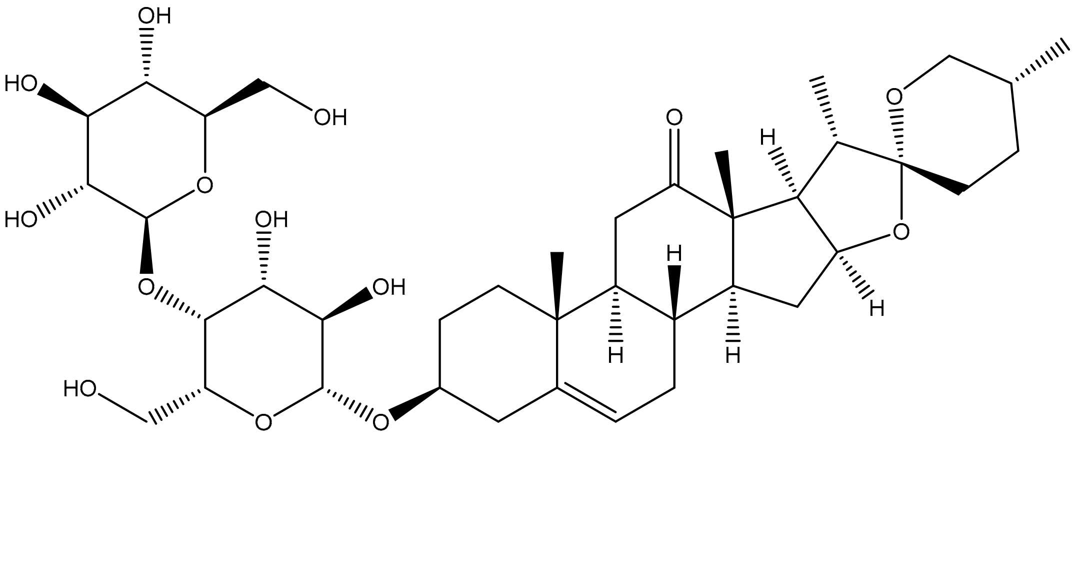

66. Tunicosaponin J 34383-24-9

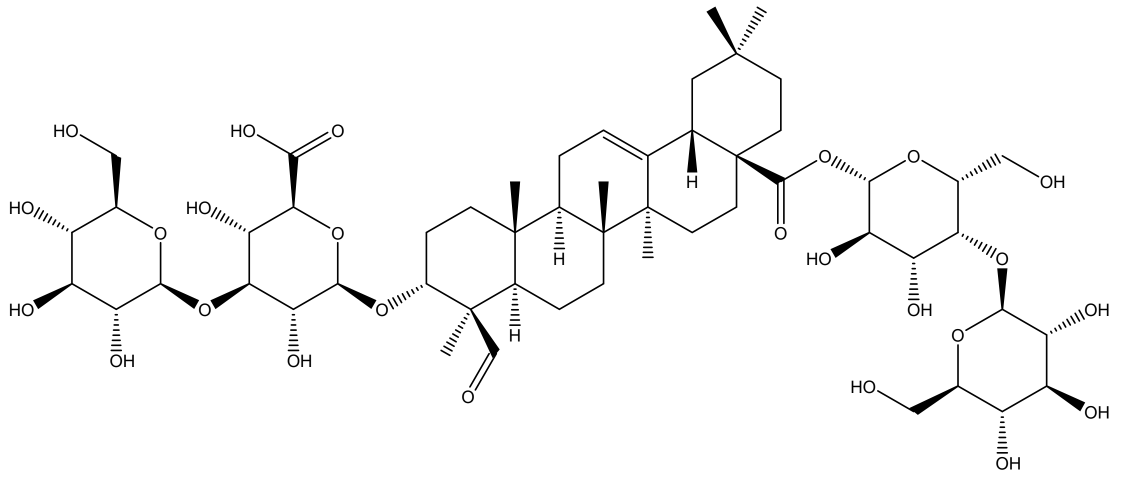

67. QUDA-(Glc)-(Glc-Glc) 210426-59-8

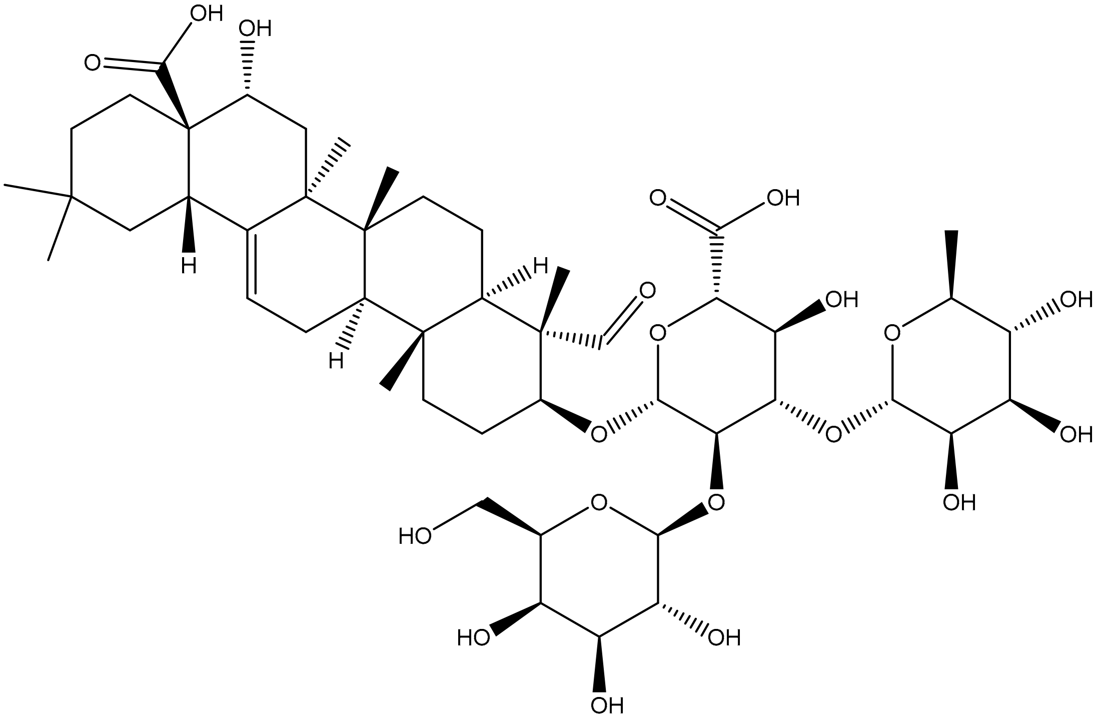

68. QUDA-Glc-Glc 138267-98-8

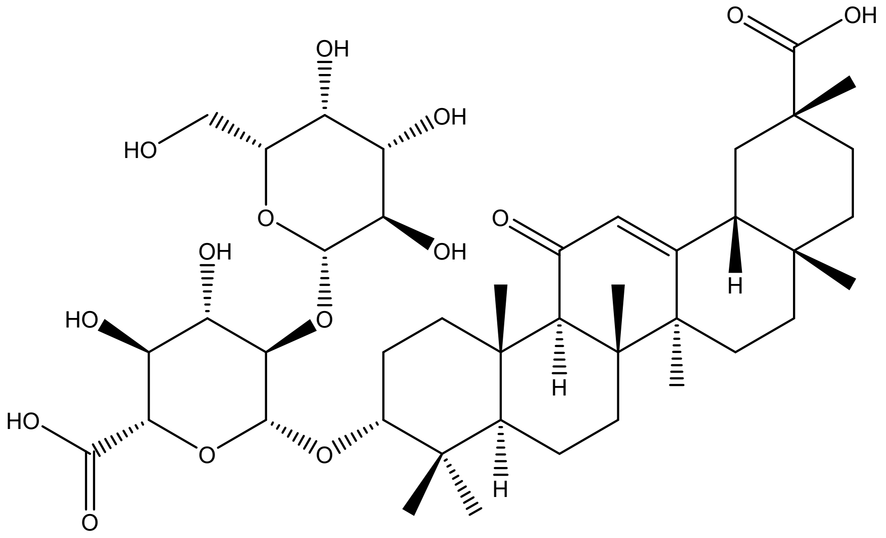

69. Silenin C PubChem CID 10350888

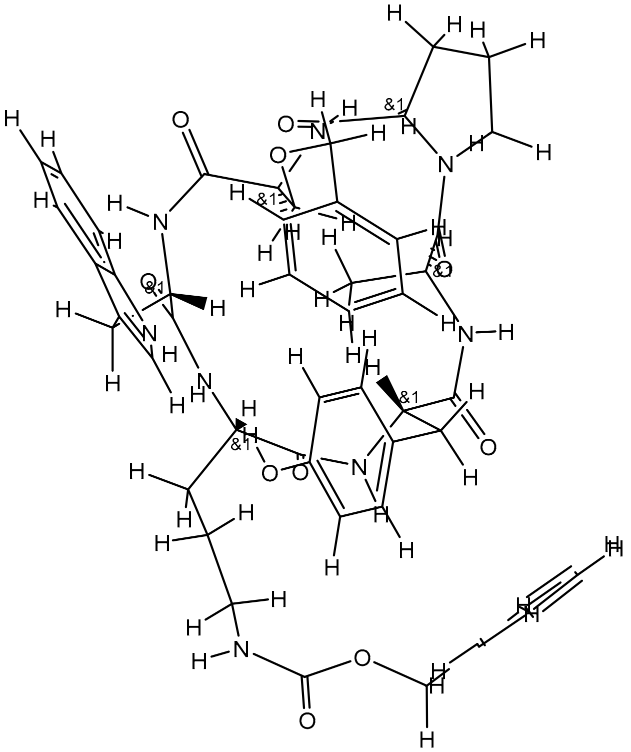

70. viticosterone E 22-O-benzoate 118201-53-9

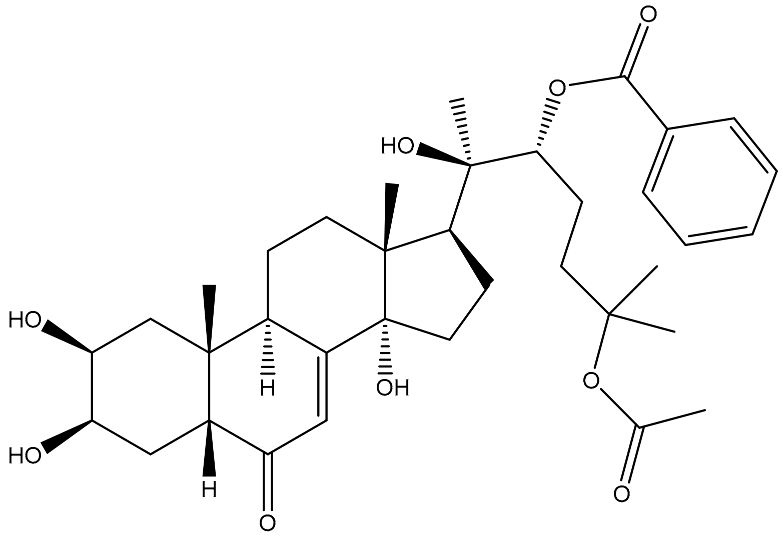

71. Sinocrassuloside X PubChem CID 73802841

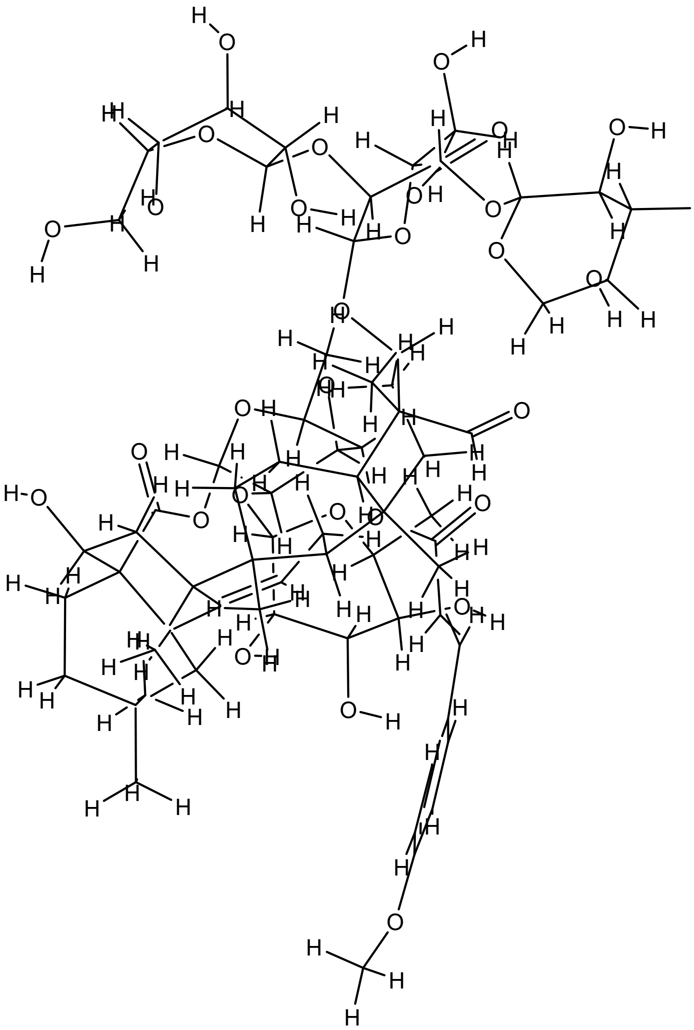

72. Quillaic acid 1309931-92-7

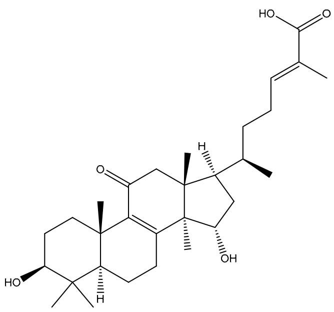

73. Ameroside F 186464-74-4

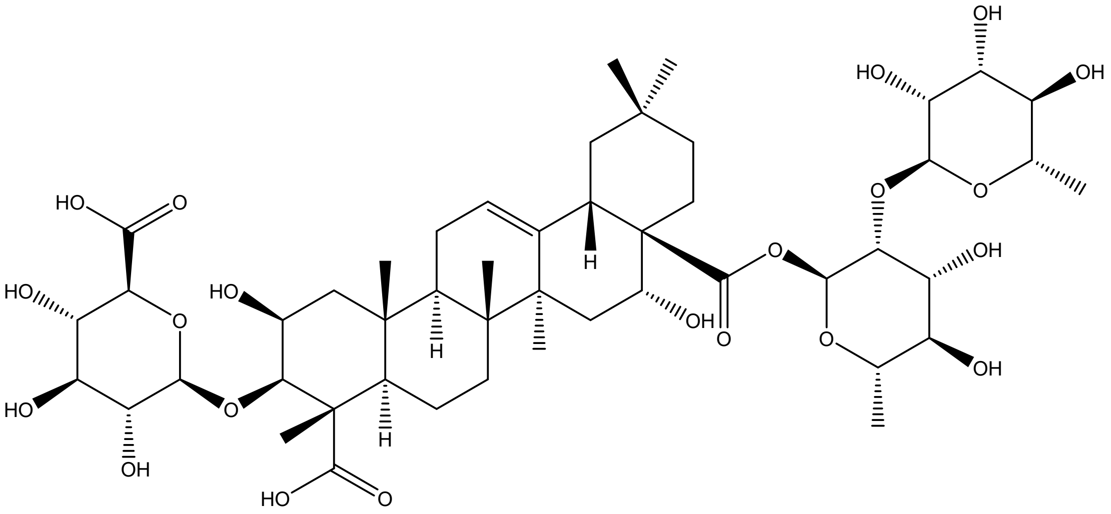

74. siliendine D 2416132-70-0

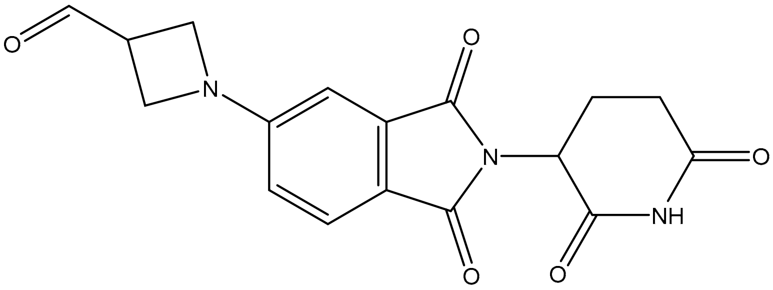

75. CanthosideC 137319-13-2

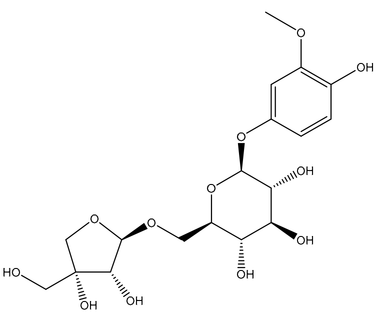

76. Armeroside C 141888-76-8

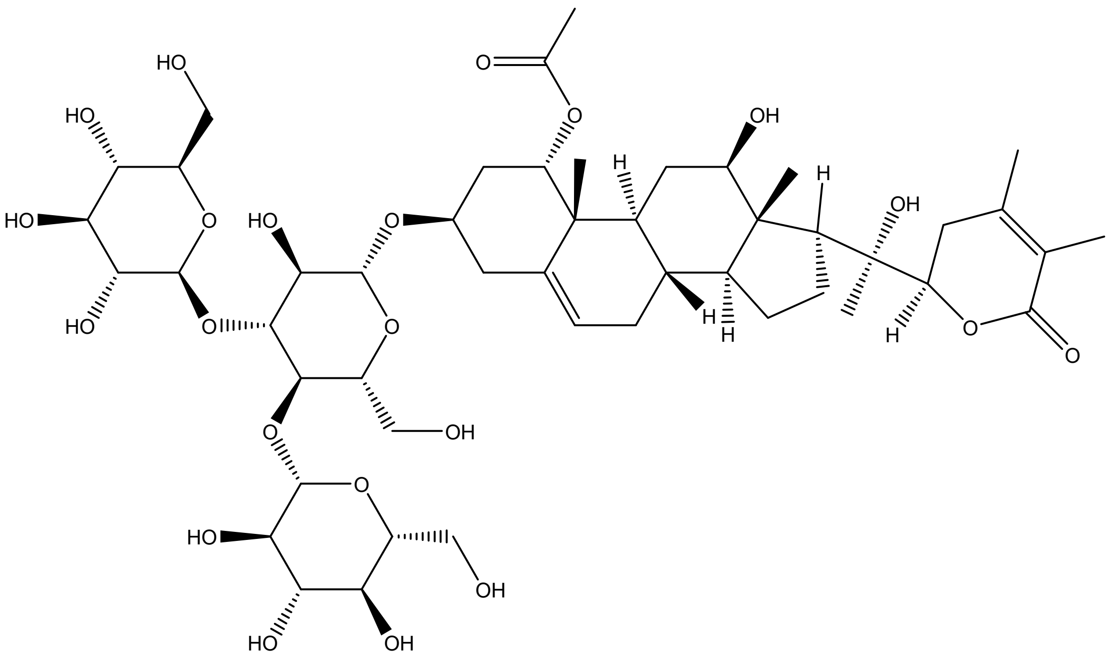

77. Silenin B PubChem CID 168983613

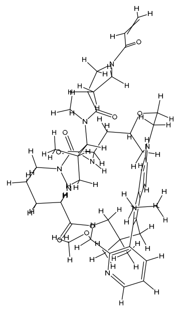

78. 2-Methylchromone 5751-48-4

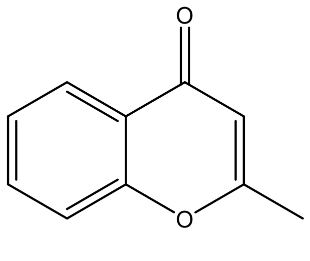

Supplement: Supplementary file 1 [file molecules-29-04817-s001.zip › Supporting material S2 compounds structures and CAS numbers.pdf]
