# Supplementary material for: Comprehensive Evaluation of Quality and Differences in Silene viscidula Franch from Different Origins Based on UPLC-ZENO-Q-TOF-MS/MS Compounds Analysis and Antioxidant Capacity
Source: Molecules. 2024 Oct 11;29(20):4817. doi: 10.3390/molecules29204817 (PMC11509892; doi:10.3390/molecules29204817)
Supplement: Supplementary file 1 [file molecules-29-04817-s001.zip › Supporting material S3 The source information of the WC samples.pdf]

Table S1. Silene viscidula Franch Herb Origin Information Sheet

No	Province	Group	Towns and Counties	Longitude and Latitude
yn1				
yn2		Y1	1.Lijiang Ninglang	E:100°51'33.510" N:27°16'55.479"
yn3				
yn4				
yn5		Y2	2.Kunming Dongchuan	E:103°11'17.783" N:26°5'27.137"
yn6				
yn7				
yn8		Y3	3.Jinghong, Xishuangbanna	E:100°48'52.288" N:21°58'24.637"
yn9				
yn10	Yun Nan			
yn11		Y4	4.Kunming Xishan	E:102°37'35.189" N:24°58'25.089"
yn12				
yn13				
yn14		Y5	5.Yuxi Xinping	E:101°59'27.876" N:24°3'58.663"
yn15				
yn16				
yn17		Y6	6.Kunming Yiliang	E:103°8'43.461" N:24°55'14.988"
yn18				

No	Province	Group	Towns and Counties	Longitude and Latitude
gx1				
gx2		G1	1.Yulin Beiliu	E:110°17'28.770" N:22°50'57.507"
gx3				
gx4				
gx5		G2	2.Yulin Bobai	E:109°58'40.115" N:22°16'53.826"
gx6				
gx7				
gx8		G3	3.Yulin Yuzhou	E:110°10'7.643" N:22°38'12.820"
gx9				
gx10				
gx11	Guang Xi	G4	4.Guilin Quanzhou	E:111°4'22.142" N:25°56'12.053"
gx12				
gx13				
gx14		G5	5.Yulin Bobai	E:109°57'7.942" N:22°12'6.737"
gx15				
gx16				
gx17		G6	6.Nanning Longan	E:107°41'51.152" N:23°10'14.834"
gx18				
gx19				
gx20		G7	7.Yulin Rongxian	E:110°33'37.299" N:22°52'10.292"
gx21				

No	Province	Group	Towns and Counties	Longitude and Latitude
sc1				
sc2		S1	1.Huidong, Liangshan	E:102°49'12.661" N:26°41'1.611"
sc3				
sc4				
sc5		S2	2.Kangding, Ganzi Prefecture	E:101°57'34.062" N:30°0'8.977"
sc6				
sc7				
sc8		S3	3.Liangshan Puge	E:102°32'23.166" N:27°22'49.497"
sc9				
sc10				
sc11	Si Chuan	S4	4.Danba, Ganzi Prefecture	E:101°52'43.672" N:30°55'43.952"
sc12				
sc13				
sc14		S5	5.Meishan Dongpo	E:103°50'38.163" N:30°2'35.109"
sc15				
sc16				
sc17		S6	6.Dachuan, Dazhou	E:107°31'25.180"N:31°11'37.514"
sc18				
sc19				
sc20		S7	7.Jinchuan, Aba Prefecture	E:102°4'37.481" N:31°28'52.563"
sc21				
